# Supplementary material for: Use of ratiometrically designed nanocarrier targeting CDK4/6 and autophagy pathways for effective pancreatic cancer treatment
Source: Nat Commun. 2020 Aug 25;11:4249. doi: 10.1038/s41467-020-17996-7 (PMC7447818; doi:10.1038/s41467-020-17996-7)
Supplement: Supplementary file 1 — Supplementary information [file 41467_2020_17996_MOESM1_ESM.pdf]

Supplementary Information

Use of ratiometrically designed nanocarrier targeting CDK4/6 and autophagy pathways for effective pancreatic cancer treatment

Ying Ji *et al.*

Supplementary Figure 1. (A) Scheme illustrating the role of CDK4/6 inhibitors in the regulation of cancer cell cycle. CDK4/6 inhibitors downregulate phosphorylated Rb (pRb) and prevent the dissociation between Rb and E2F transcription factors. The absence of free E2F transcription factor leads to interrupted G1 to S phase transition and results in G1 cell cycle arrest. (B) For screening purpose, a list of human PDAC cell lines and a KPC cell line (derived from a spontaneous PDAC tumor from a transgenic $Kras^{LSL\text{-}G12D/+}Trp53^{LSL\text{-}R172H/+}Pdx1\text{-}Cre$ mouse) were assayed for Rb and pRb expression. All the human PDAC cell lines are positive for both Rb and pRb. KPC cells exhibit an extremely low Rb/pRb expression, which is in agreement with the literature[1]. Five Rb/pRb^{+} PDAC cell lines, namely PANC-1, MIA PaCa-2, BxPC-3, HPAF-II and ASPC-1, are included in our investigation.

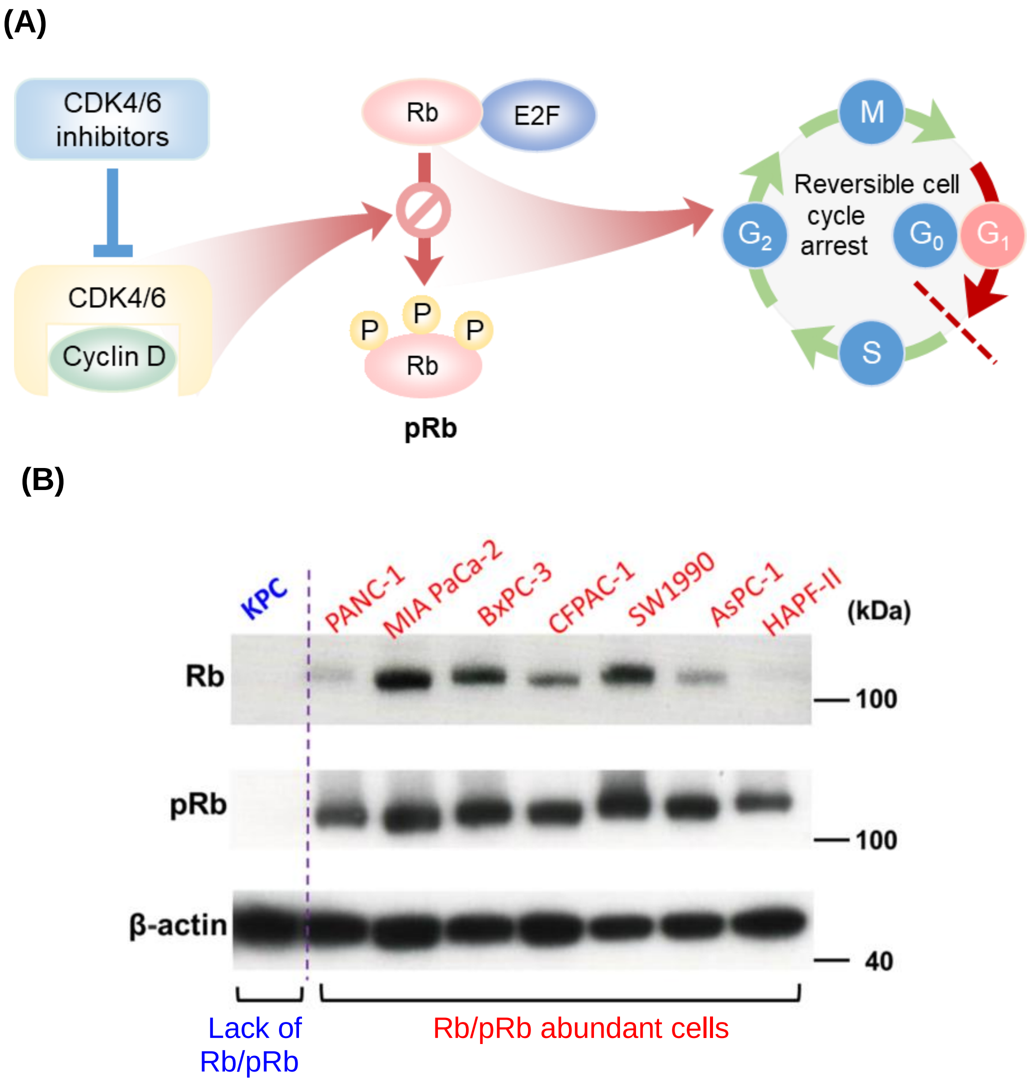

Supplementary Figure 2. PAL/HCQ free drug combinations synergize in additional PDAC cell lines. PDAC cell lines were incubated with a series of free PAL/HCQ at various combinatory molar ratios. Treatment of each concentration was tested in triplicate wells in a 96-well BrdU proliferation assay (n=3). Half maximal inhibitory concentration (IC_{50}) and combination index (CI) were calculated for each combination ratio *via* CompuSyn software. Similar to PANC-1 data, PAL/HCQ at 1:5 shows strong synergy with CI index <0.5. Source data are provided in the Source Data file.

MIA PaCa-2

Free PAL alone IC_{50} =6.8 µM

Free HCQ alone IC_{50} >50 µM

IC_{50} (µM)	Free PAL/HCQ ratio 10/1	2/1	1/1	1/5	1/10
PAL (µM)	11.2	7.7	3.4	1.8	1.1
HCQ (µM)	1.2	3.9	3.4	9.1	10.7
CI	1.47	1.02	0.47	0.27	0.22

BxPC-3

Free PAL alone IC_{50} =51.8 µM

Free HCQ alone IC_{50} =33.2 µM

IC_{50} (µM)	Free PAL/HCQ ratio 10/1	2/1	1/1	1/5	1/10
PAL (µM)	42.6	62.8	22.3	1.9	1.9
HCQ (µM)	4.3	31.4	22.3	9.6	19.6
CI	0.95	2.16	1.10	0.32	0.63

AsPC-1

Free PAL alone IC_{50} =194.8 µM

Free HCQ alone IC_{50} =299.2 µM

IC_{50} (µM)	Free PAL/HCQ ratio 10/1	2/1	1/1	1/5	1/10
PAL (µM)	640.7	434.8	47.9	14.7	17.3
HCQ (µM)	64.1	217.4	47.9	73.6	173.1
CI	3.50	2.96	0.41	0.32	0.67

HPAF-II

Free PAL alone IC_{50} =5.68 µM

Free HCQ alone IC_{50} =312.5 µM

IC_{50} (µM)	Free PAL/HCQ ratio 10/1	2/1	1/1	1/5	1/10
PAL (µM)	4.8	5.9	3.9	2.5	2.5
HCQ (µM)	0.47	2.9	3.9	12.5	25.2
CI	0.84	1.04	0.70	0.48	0.52

Supplementary Figure 3. PAL/HCQ treated PANC-1 cells are subjected to G1 cell cycle arrest. PANC-1 cells were incubated with free PAL/HCQ (PAL=2.5 μM, HCQ=12.5 μM; PAL/HCQ=1:5 molar ratio) for 72 h. PANC-1 cells incubated with free PAL alone or free HCQ alone at equivalent concentration were also tested. Cells were fixed with 4% paraformaldehyde and stained with Tali™ cell cycle kit (Thermo Fisher). Cell cycle was determined on a flow cytometer (LSRII, Becton Dickinson) and cell population in G1, S and G2/M phase was analyzed by FlowJo software (v7.6.1) from cell samples in triplicate (n=3). Data represents mean ± SD. Statistical difference was evaluated by one-way ANOVA followed by Tukey's post hoc test (*$p<0.05$). Both PAL ($p=0.00063$) and PAL/HCQ combination ($p=0.00071$) induce significant G1 cell cycle arrest compared to control. Source data are provided as a Source Data file.

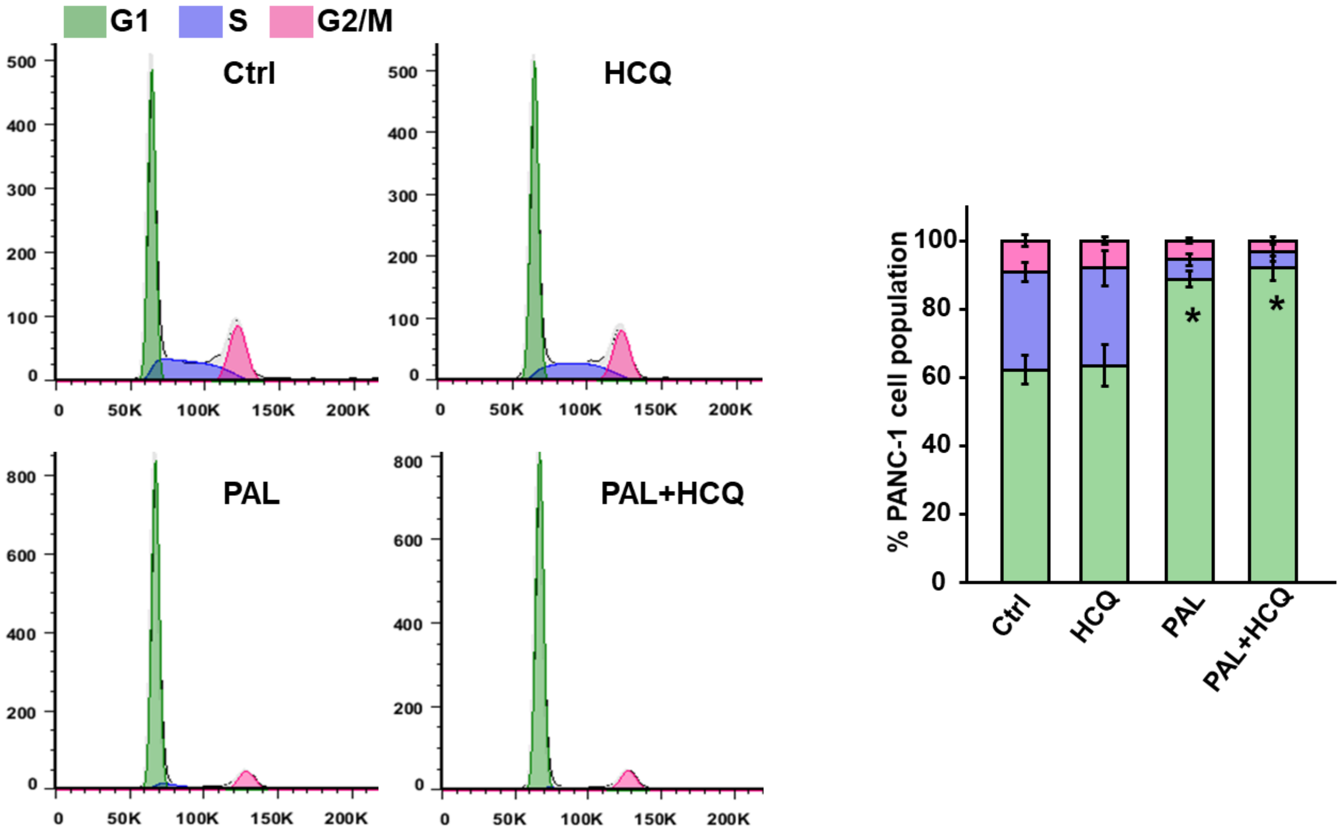

Supplementary Figure 4. Combination of PAL/HCQ free drug demonstrates synergized efficacy in MIA PaCa-2 cells, by co-targeting CDK4/6 mediated cell cycle progression and autophagy pathway. MIA PaCa-2 cells were incubated with free PAL/HCQ combination (1:5 molar ratio, PAL=2.5 μM, HCQ=12.5 μM) for 72 h and subjected to the following assays to elaborate the associated biological events. MIA PaCa-2 cells treated by free PAL alone or free HCQ alone were also compared. One of two repetitions with similar results is shown here. **(A)** Crystal violet staining of MIA PaCa-2 cells visualizes the synergistic anti-proliferation effect by PAL/HCQ pair. **(B)** Immunofluorescence staining of Ki67 proliferation marker (red) and nuclei (blue); scale bar=20 μm. Quantification of Ki67 expression was obtained by determining the percentage of Ki67 positive cells in each ROI (n=6). Data represent mean ± SD, statistical difference was evaluated by one-way ANOVA followed by Tukey's post hoc test (**p*<0.05). Free PAL/HCQ combination (1:5 molar ratio) displays significantly enhanced anti-proliferative effect compared to PAL mono-treatment (*p*=0.021). **(C)** Immunofluorescence staining of pRb (red) demonstrates that free PAL/HCQ combination (1:5 molar ratio) effectively reduces the pRb expression. Scale bar=20 μm. **(D)** GFP-RFP-LC3B reporter assay demonstrates that MIA PaCa-2 cells treated by PAL/HCQ combination exhibit the most distinct accumulation of autophagosome (both GFP and RFP puncta) with impaired fusion into autolysosomes (RFP-only puncta). sQuantification of GFP-RFP-LC3B was obtained by determining the total number of green or red puncta per cell (n=6). Data represent mean ± SD. Statistical difference was evaluated by one-way ANOVA followed by Tukey's post hoc test (**p*<0.05). The level of RFP-LC3B puncta is significantly enhanced over GFP-puncta in PAL treated cells ($p=1.06 \times 10^{-7}$), demonstrating the accumulation of autolysosomes. Scale bar=20 μm. **(E)** CellROX staining of reactive oxygen species (ROS) suggests that PAL/HCQ combination induces the highest level of intracellular ROS accumulation. Source data are provided as a Source Data file.

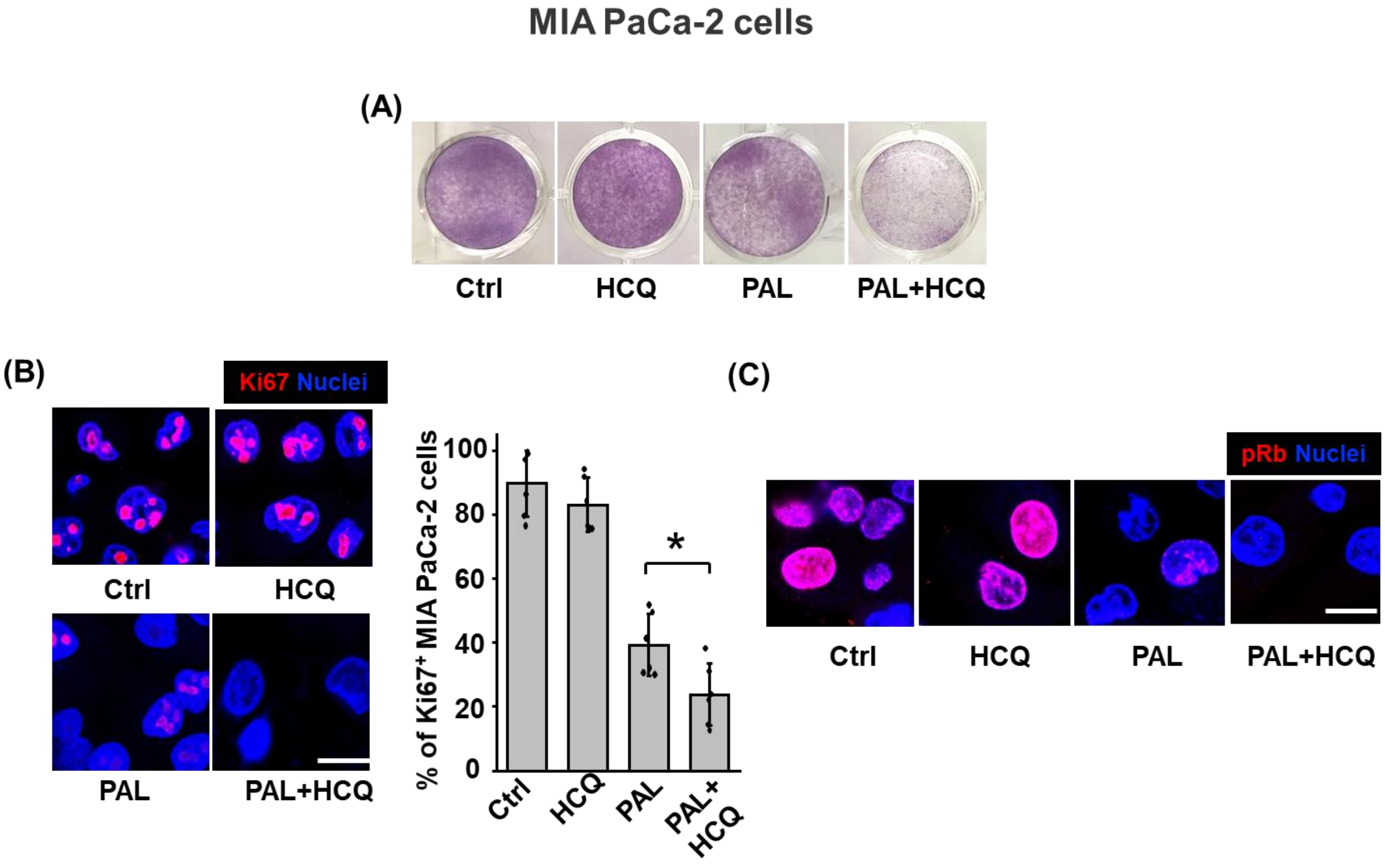

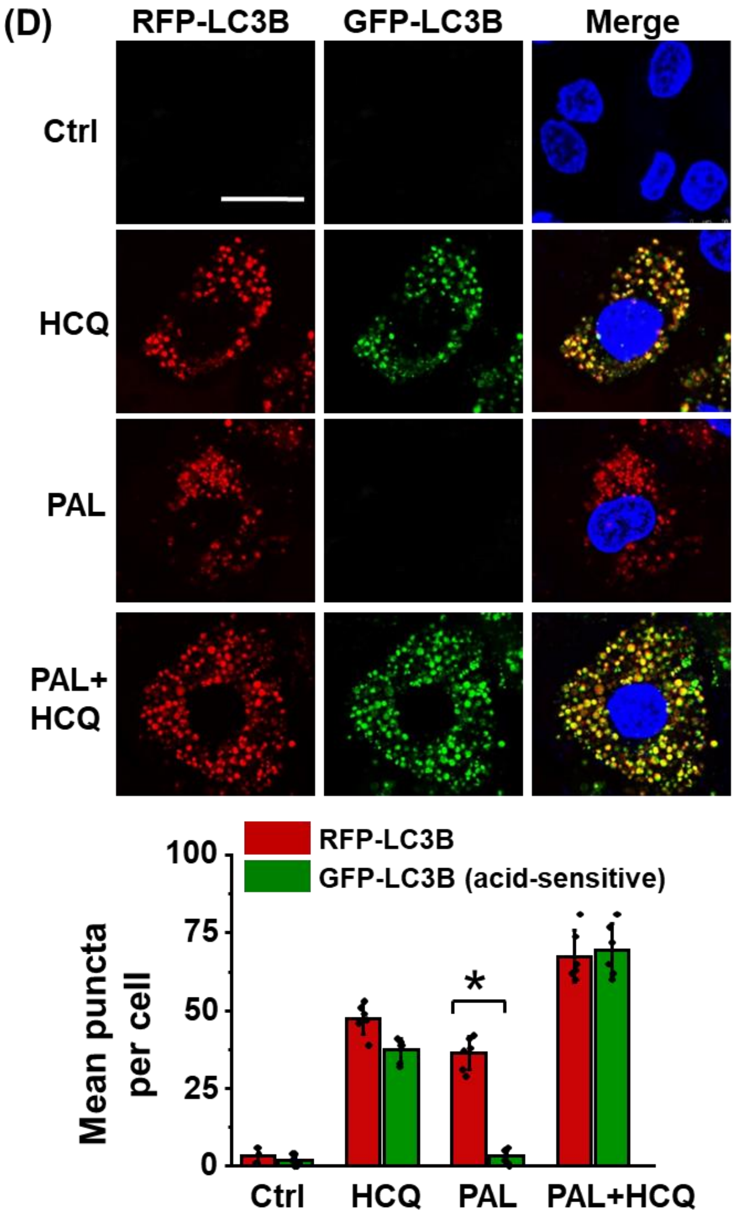

(D)
RFP-LC3B
GFP-LC3B
Merge
Ctrl
HCQ
PAL
PAL+
HCQ
RFP-LC3B
GFP-LC3B (acid-sensitive)
100
75
50
25
0
Mean puncta per cell
*
Ctrl
HCQ
PAL
PAL+HCQ

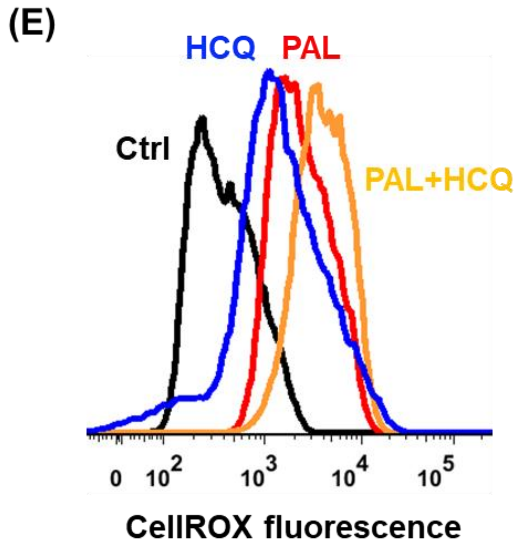

(E)
HCQ
PAL
Ctrl
PAL+HCQ
0
10^2
10^3
10^4
10^5
CellROX fluorescence

Supplementary Figure 5. General considerations for remote loading of ratiometrically-designed weak base drugs. The general characteristics of these cargos should include the following physiochemical properties: ***(i)*** compounds that include primary, secondary or tertiary amine(s); ***(ii)*** pKa<11 to allow protonation and entrapment inside the lipid coat; ***(iii)*** water solubility ranging from around 5 to 25 mg/mL and amphipathic characteristic that allows diffusion across the lipid bilayer; ***(iv)*** an octanol/water partition coefficient or log P value of -3.0 to 3.0; ***(v)*** suitable molecular weight with a geometric size less than MSNP pore size (2-8 nm) to allow entry into the MSNP pores. In addition, the drug pair should ideally exhibit similar range of pKa and solubility, and can be co-imported by the use of a single proton-releasing trapping agent. Moreover, for drug co-import, the loading conditions such as pH and buffer solutions, should be compatible for both cargos.

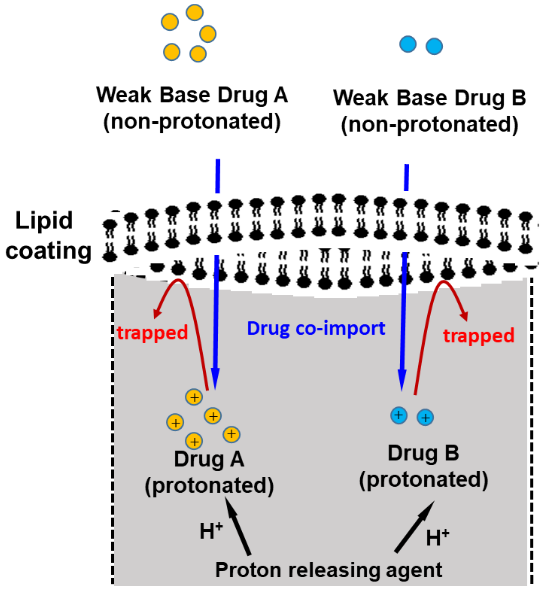

Considerations of weak base remote loading

(i) Organic molecules that include primary, secondary or tertiary amine(s);
(ii) pKa <11 to allow protonation and entrapment inside the lipid coat;
(iii) Water solubility ranging from around 5~25 mg/mL and amphipathic characteristics that allow diffusion across the lipid coat;
(iv) Octanol/water partition coefficient or log P value of -3.0 to 3.0;
(v) Suitable molecular weight with a geometric size less than MSNP pore size (2-8 nm) to allow entry into the MSNP pores.

Considerations of ratiometric co-import

(vi) Drug A and Drug B should have similar pKa and solubility values that can be imported into particle at comparable efficiency
(vii) For ease of drug co-import, use of a single trapping agent is preferred.

Supplementary Figure 6. Optimization of loading condition for co-encapsulation of PAL and HCQ *via* a simultaneous remote loading. **(A)** Iterative optimization was performed and a list of parameters (pH, incubation time, MSNP/HCQ/PAL feed ratios) were systemically explored. While both PAL and HCQ are suitable candidates for remote loading, loading optimization is a necessary step because the minor difference between each API requires additional consideration. Take pH for example, we find that the solubility of PAL is more pH-sensitive than HCQ. At pH 7.4, the solubility of PAL is negatively impacted so that the PAL diffusion across lipid-bilayer is hindered (loading content < 3 wt%). It is also important to control the pH greater than 5.0, otherwise the majority of PAL and HCQ are protonated outside the particle. This prompted us to use 5 mM HEPES + 5% dextrose at pH 6.5, at which a balanced solubility, protonation state and pH gradient have been achieved. The optimized loading condition is identified as pH=6.5, 30 min incubation with PAL and HCQ, with the nanoparticle / PAL feed ratio ranging from 1:0.05 to 1:0.25 (wt/wt) and nanoparticle/HCQ ratio ranging from 1:0.05 to 1:0.5 (wt/wt), which correlate to the PAL feed concentration range (0.25 to 2.5 mg/mL) and HCQ feed concentration range (0.25 to 5 mg/mL). **(B)** To optimize the loading condition, drug loading content and encapsulation efficiency were characterized by HPLC. Here we demonstrate the representative HPLC detection of PAL and HCQ content in the co-delivery NP. PAL and HCQ in co-delivery NP was extracted *via* acidic methanol solution as described in the method section. The extracted drug containing supernatants were filtrated through 0.22 μm filters for HPLC analysis on PerkinElmer Altus A-10 HPLC System with Brownlee SPP 2.7 μm C18 column, followed by UV detection at 340 nm. The mobile phase contained 3% triethylammonium acetate aqueous solution (pH 5.6) and acetonitrile (73:27, v/v) at a flow rate of 0.2 mL/min. The typical retention time is identified as 2.4 min for HCQ and 5.9 min for PAL, respectively.

(A)

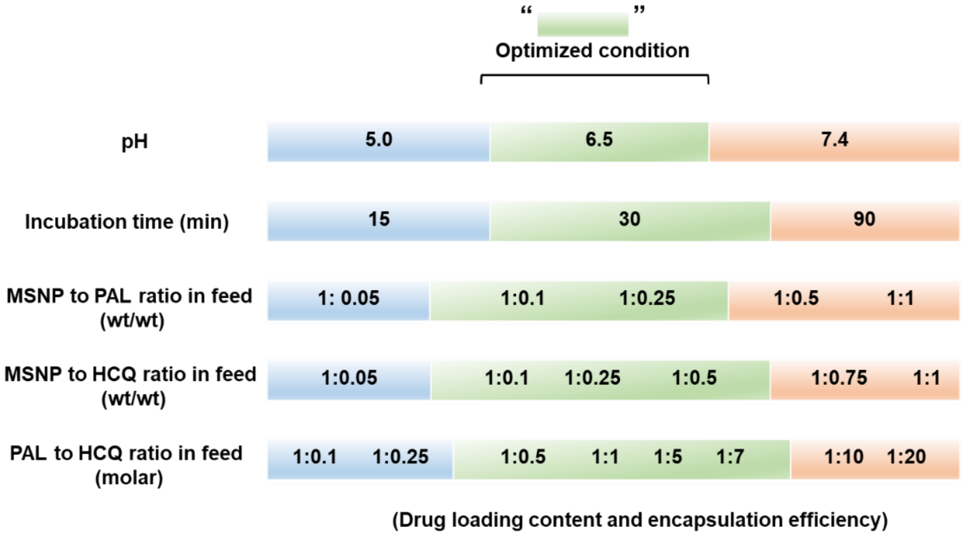

(B)

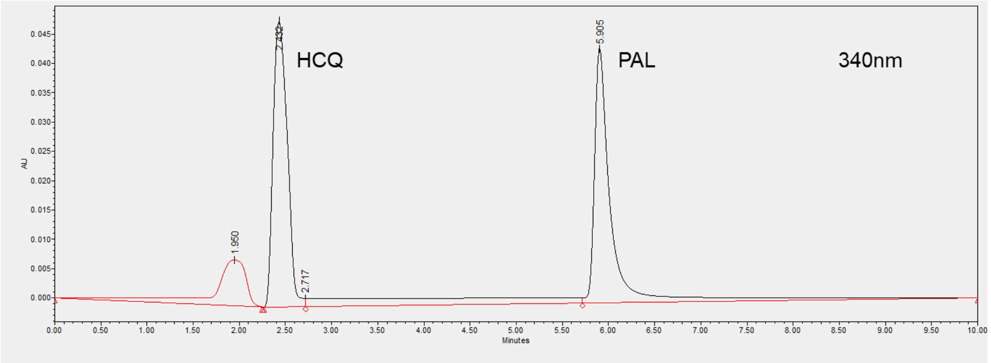

Supplementary Figure 7. *In vitro* study for the co-delivery NP in PANC-1 cells. PANC-1 cells were incubated with co-delivery NP for 72 h. This equals to PAL concentration of 2.5 μM and HCQ concentration of 10.8 μM, respectively. Nanoparticles loaded with PAL-alone (PAL NP) or HCQ-alone (HCQ NP) were also included as controls. One of three repetitions with similar results is shown here. **(A)** Immunofluorescence staining of proliferative marker Ki67 to further visualize the synergized anti-proliferative effect by co-delivery NP in Figure 2F. Scale bar=20 μm. Quantification of Ki67 expression was obtained by determining the % of Ki67 positive cells in each ROI (n=6). Data represent mean ± SD. Statistical difference was evaluated by one-way ANOVA followed by Tukey's post hoc test (*$p<0.05$). Co-delivery NP lead to significantly enhanced anti-proliferative effect compared to PAL NP ($p=5.7\times10^{-4}$). **(B)** Representative cell cycle analysis for Figure 2G detected by flow cytometry. **(C)** Immunofluorescence staining of pRb (red) and nuclei (blue) suggests that co-delivery NP effectively inhibit the phosphorylation of Rb, which is dominant for cell cycle regulation. Scale bar=10 μm. **(D)** Supplementary data for GFP-RFP-LC3B reporter assay in Figure 2J. Quantification of GFP-RFP-LC3B was obtained by determining the total number of green or red puncta per cell (n=6). Scale bar=20 μm. Visualization and quantification of autophagosomes (both GPF and RFP puncta) and autolysosomes (RFP-only puncta) indicates that co-delivery NP block the autophagosome-lysosome fusion and result in significant accumulation of autophagosomes. This contrasts to mono-treatment by PAL NP, which lead to significantly enhanced level of autolysosomes ($p=2.35\times10^{-6}$), suggesting the intact autophagy flux. **(E)** Immunofluorescence staining of p62 protein (red) reveals distinct inhibition on p62 degradation by co-delivery NP as compared to PAL NP at 72 h, indicating the blockage on autophagy flux by co-delivery NP. Scale bar=10 μm. Source data are provided as a Source Data file.

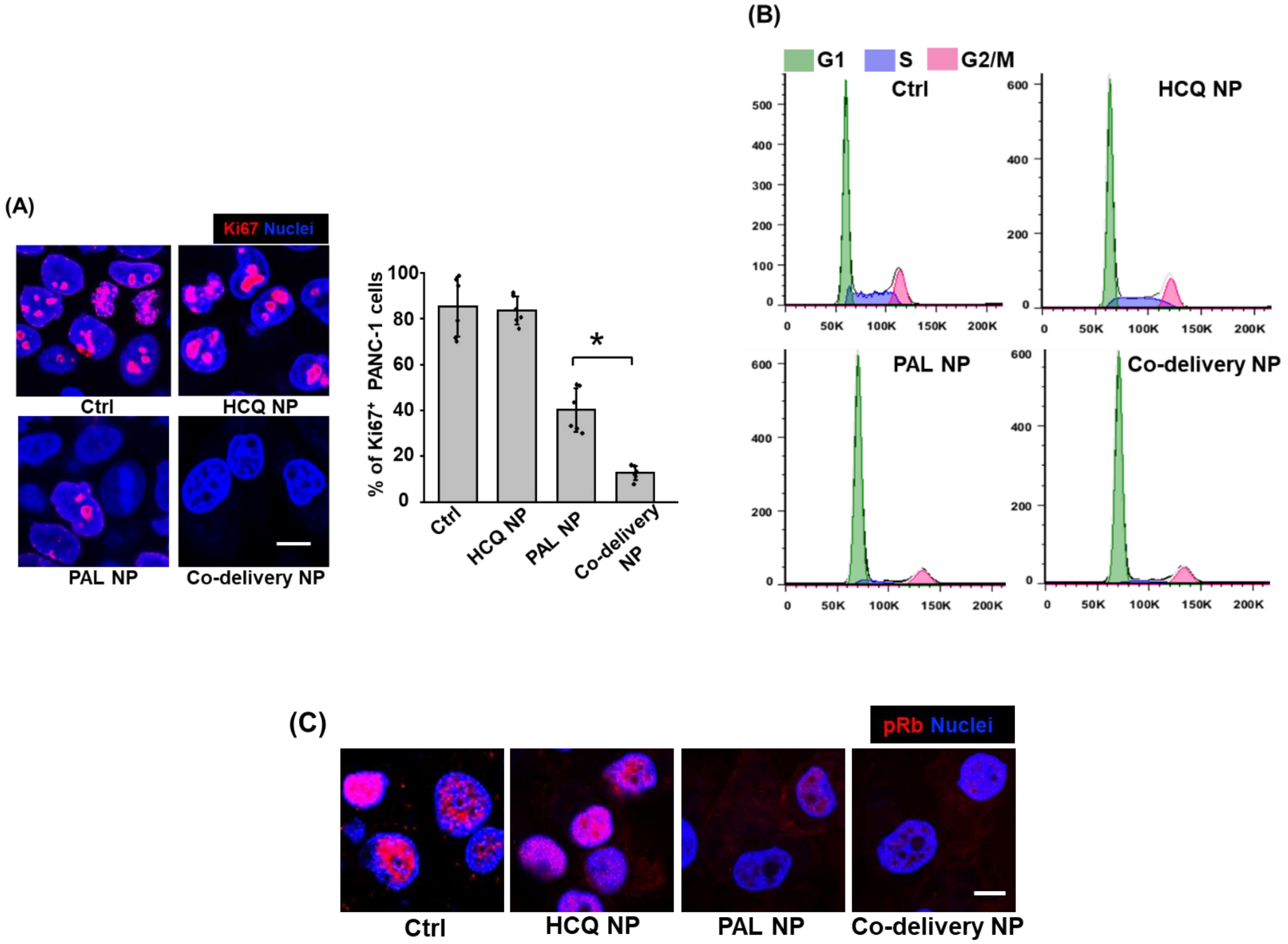

(D)

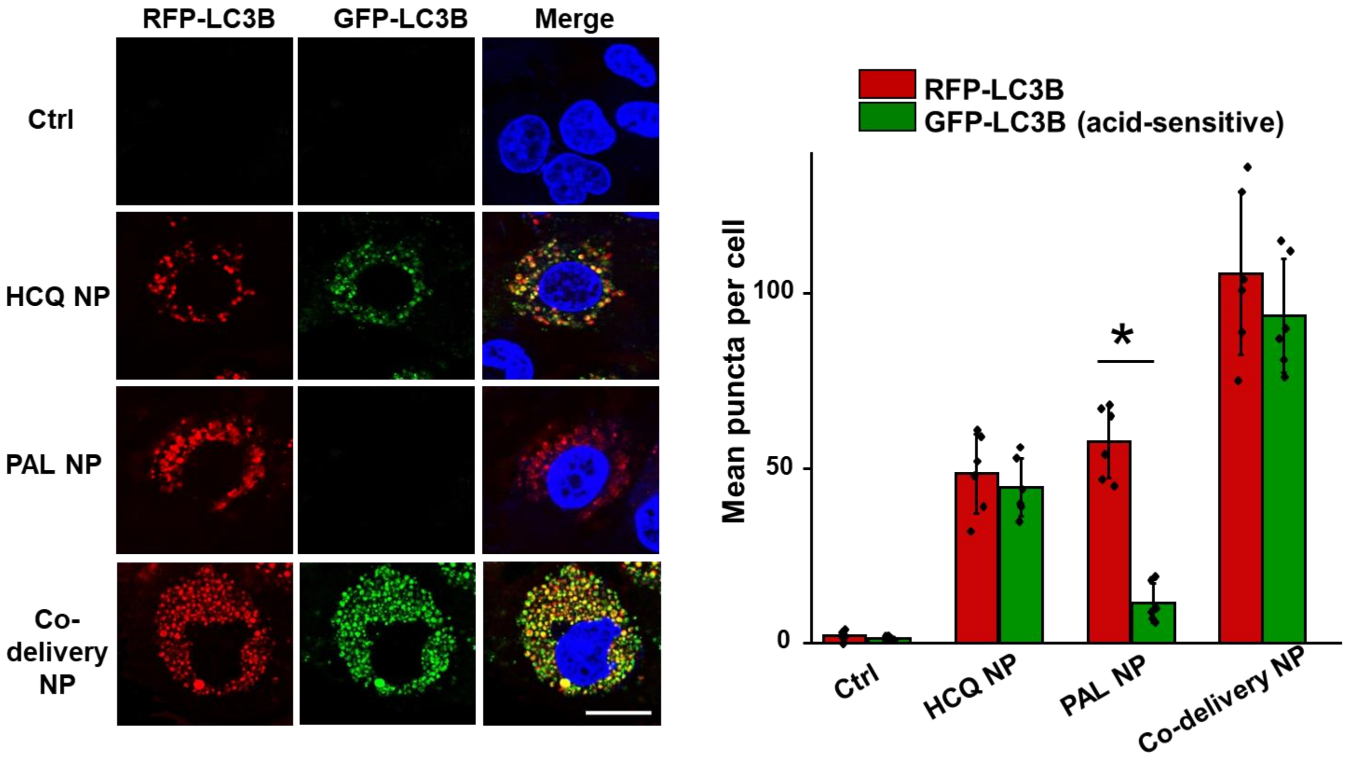

RFP-LC3B
GFP-LC3B
Merge
Ctrl
HCQ NP
PAL NP
Co-delivery NP
RFP-LC3B
GFP-LC3B (acid-sensitive)
Mean puncta per cell
100
50
0
*
Ctrl
HCQ NP
PAL NP
Co-delivery NP

(E)

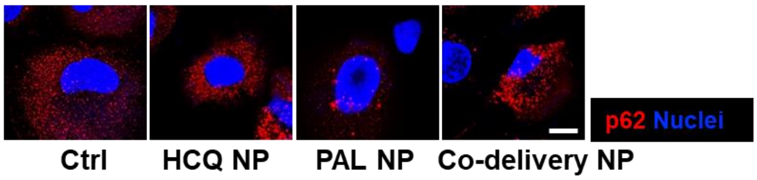

p62 Nuclei
Ctrl
HCQ NP
PAL NP
Co-delivery NP

Supplementary Figure 8. An example of gating strategy for the flow cytometry analysis of cell cycle and ROS production in Figures 1F, 2G, 2K, Supplementary Figure 3 and 4E. (A) For cell cycle analysis, SSC-A/FFC-A were used to gate live cells, and the PE channel (for detection of propidium iodide) were analyzed with PE-A/PE-W to gate the single cells. Cell cycle analysis were then performed in the population of single cells. (B) For ROS detection, SSC-A/FFC-A and SSC-W/SSC-A were used to gate the single cells; SYTOX Red dead cells stain (detected with APC channel) was used to gate the live cells. ROS production (stained with CellROX green) was detected with Alexa Fluor-488 channel.

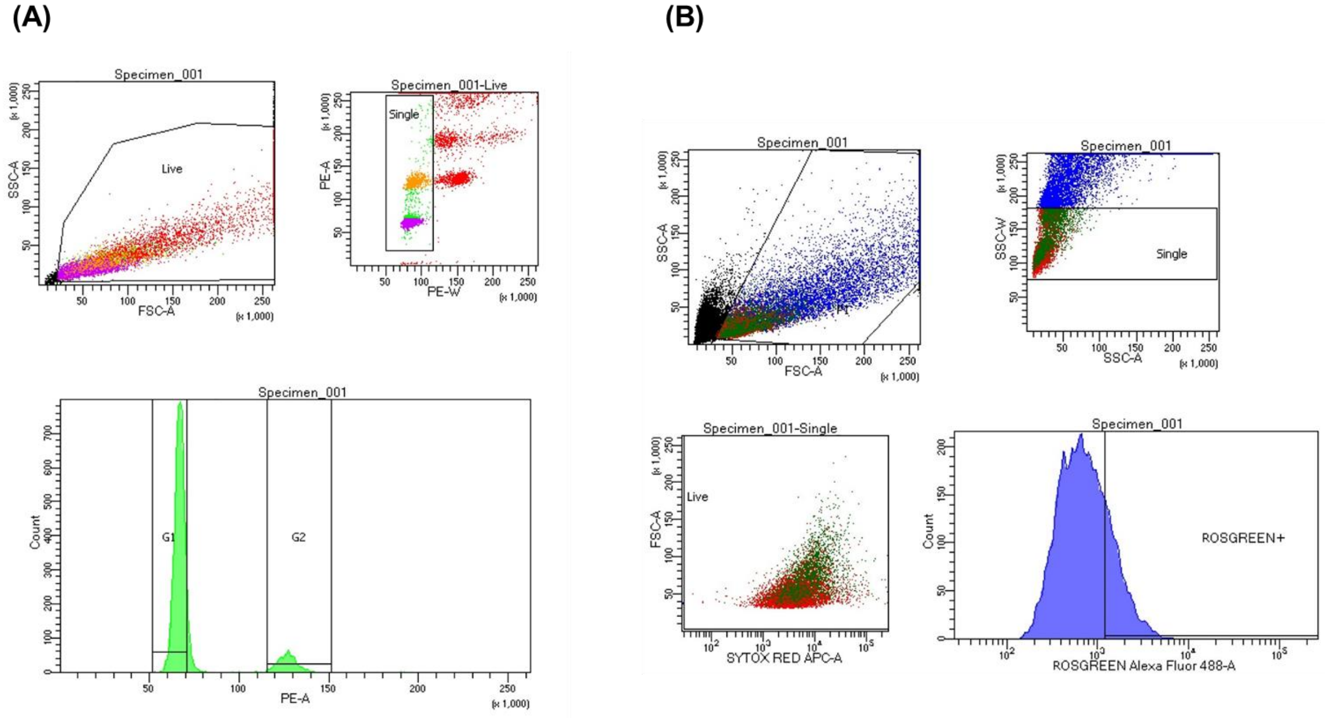

Supplementary Figure 9. Western blot study of Ki67, p62, LC3B expression in PANC-1 cells treated with the co-delivery NP. PANC-1 cells were incubated with co-delivery NP, HCQ NP or PAL NP for 72 h, with equivalent concentration of PAL (2.5 μM) and HCQ (10.8 μM). Western blot analysis was performed and one of two repetitions with similar results is shown here. The protein levels were quantified with ImageJ software (v1.5.2) and normalized to control. Western blot detection of Ki67 demonstrates significant anti-proliferative effect by PAL NP and co-delivery NP. Compared to PAL NP, co-delivery NP lead to enhanced LC3II/LC3I ratio and reduced p62 degradation, which demonstrates the inhibition of autophagy flux (supplementary to Figure 2J and Supplementary Figure 7). Uncropped gel images are provided as a Source Data file.

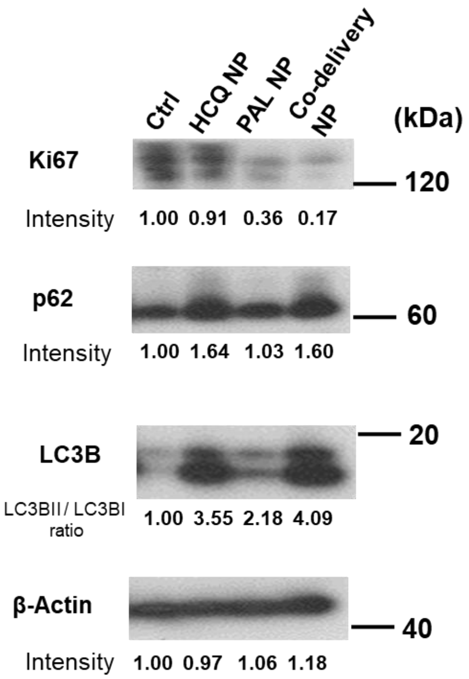

Supplementary Figure 10. Representative UPLC-MS detection of the concentration of PAL and HCQ in plasma 24 h post i.v. injection of co-delivery NP or free drug mixture. After separation of the plasma fraction, the drug was extracted in an acidic methanol solution (0.1 mol/L phosphoric acid/methanol, 1:4 v/v). Detailed sample preparation was described in the method section. The PAL and HCQ concentrations were measured by UPLC-MS (Waters LCT Premier ESI). At 24 h, free PAL is hardly detected in plasma, while residual free HCQ is still observed. This contrasts to co-delivery NP, by which significantly enhanced plasma concentrations of both PAL and HCQ are detected, due to the advantageous circulatory properties as demonstrated in Figure 3A.

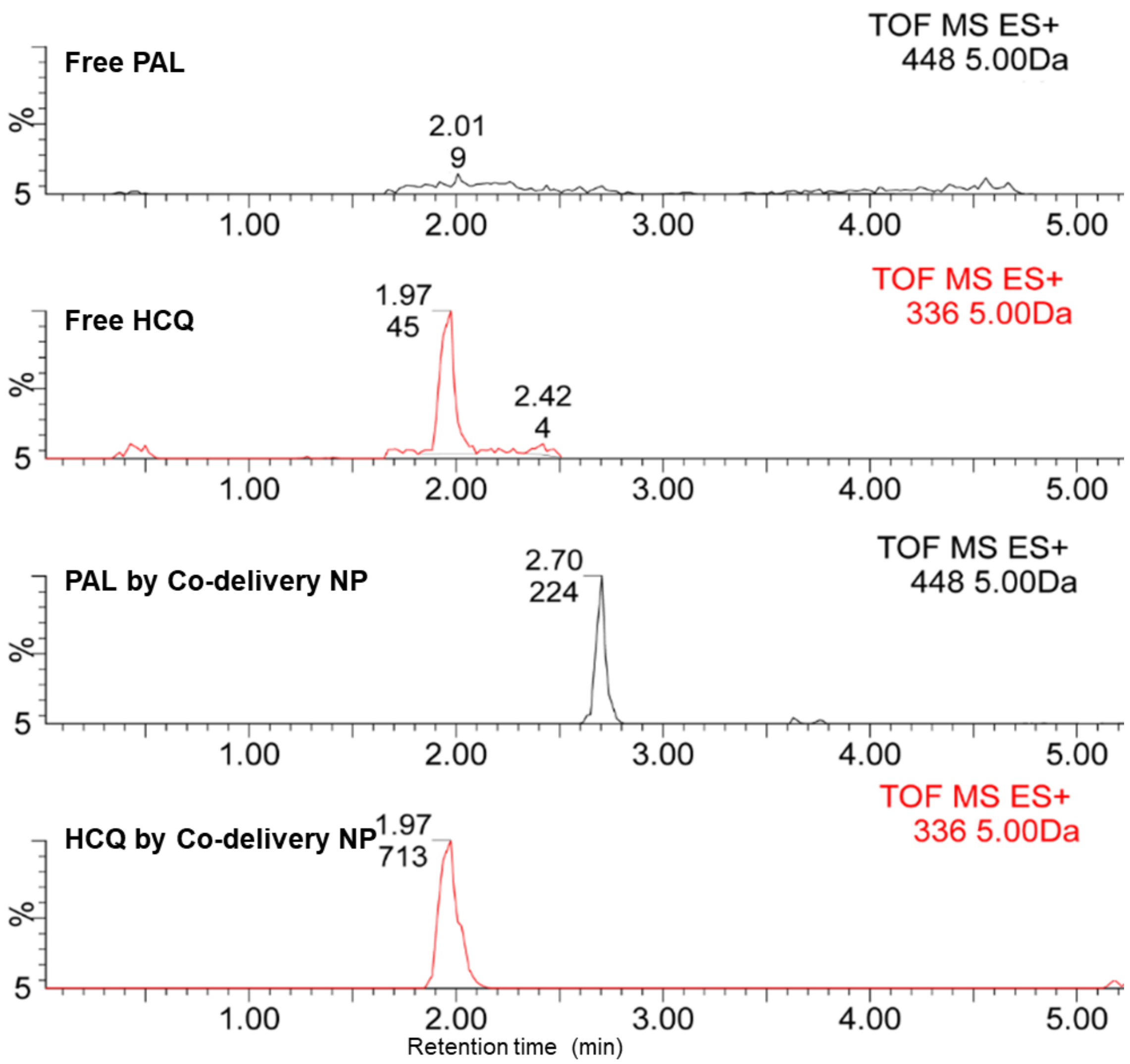

Supplementary Figure 11. Representative IVIS images to display the complete monitoring of tumor development in orthotopic PANC-1 xenograft mice model in Figure 4D. Briefly, mice received 5 i.v. injections of co-delivery NP (210 mg particles/kg/injection), free PAL/HCQ pair, PAL NP, HCQ NP or saline control, with an equivalent PAL dose of 10 mg/kg/injection and a HCQ dose of 33 mg/kg/injection. Bioluminescence based detection of PANC-1 tumor signal was recorded every 5 days from day 1 to day 30. Both live imaging and the *ex vivo* imaging at the endpoint suggest that co-delivery NP elicit significantly enhanced anti-tumor effect as compared to either single drug laden NP or free drug mixture. Data were obtained from one set of independent experiment without repetition.

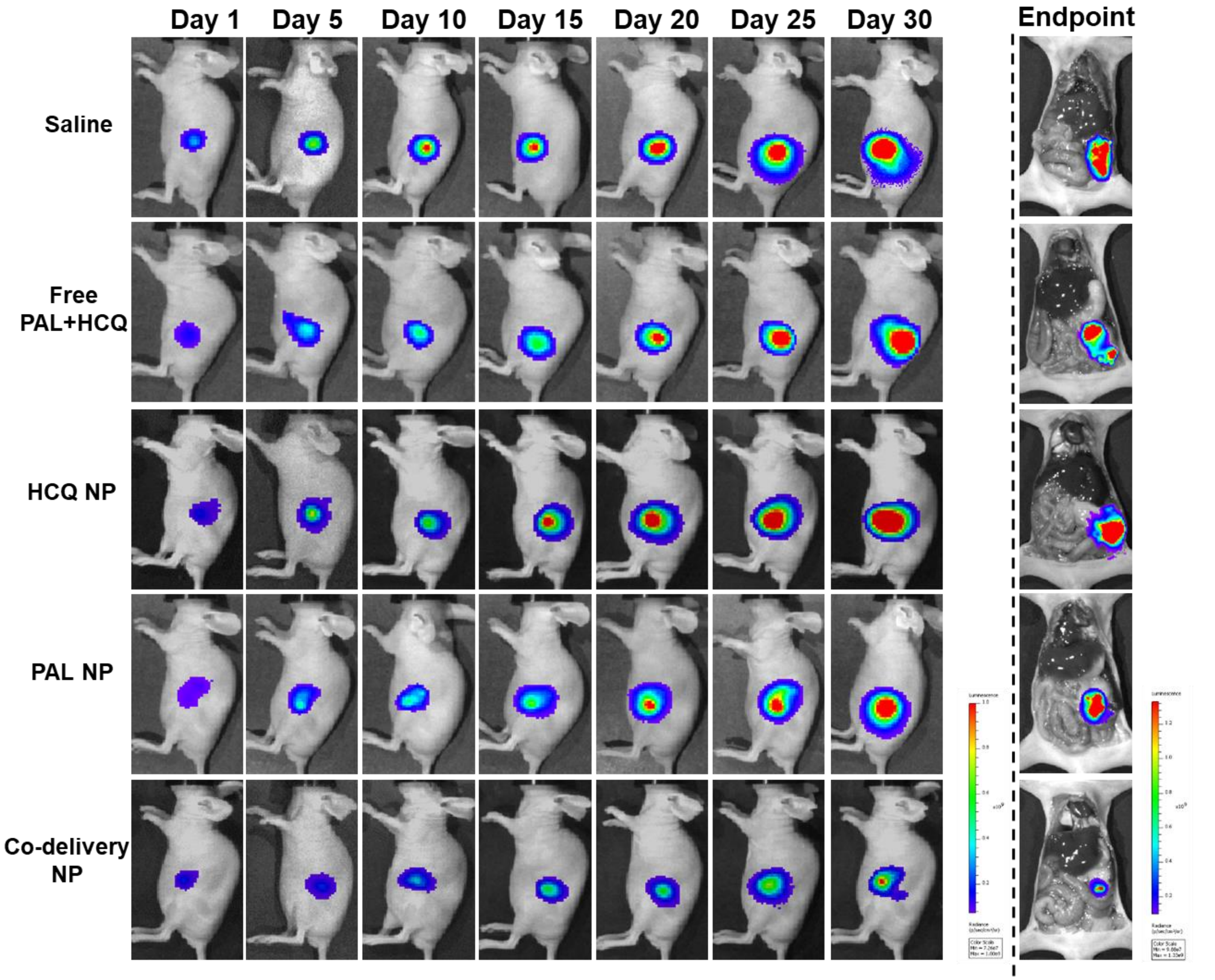

Supplementary Figure 12. *In vivo* safety study of co-delivery NP by whole blood counts, blood chemistry test and histological examination. Blood samples and major organs were harvested from the same efficacy study as we described in Figure 4D. Data were obtained from one set of independent experiment without repetition (n=3). **(A)** Complete blood counts including white blood cells (WBC), red blood cells (RBC), platelets (PLT), neutrophil (NE) and lymphocytes (LY). **(B)** RBC differential count and **(C)** WBC differential count. Data represent mean ± SD, statistical difference was evaluated by one-way ANOVA followed by Tukey's post hoc test. No statistical significance is observed between different groups. The results indicate no detectable neutropenia or leukopenia in the group receiving co-delivery NP, compared to control group. **(D)** Blood biochemistry analysis (AST, ALT, ALP, BUN, creatinine, DBILI, TBILI, LDH, TP, and CK). The results indicate no damage to liver or kidneys, as well as no sign of dysfunction in other organs. **(E)** H&E staining of major organs including heart, liver, lung spleen, kidneys, sternum and intestine, demonstrates the absence of pathological abnormalities by co-delivery NP. Scale bar=200 μm. Source data are provided as a Source Data file.

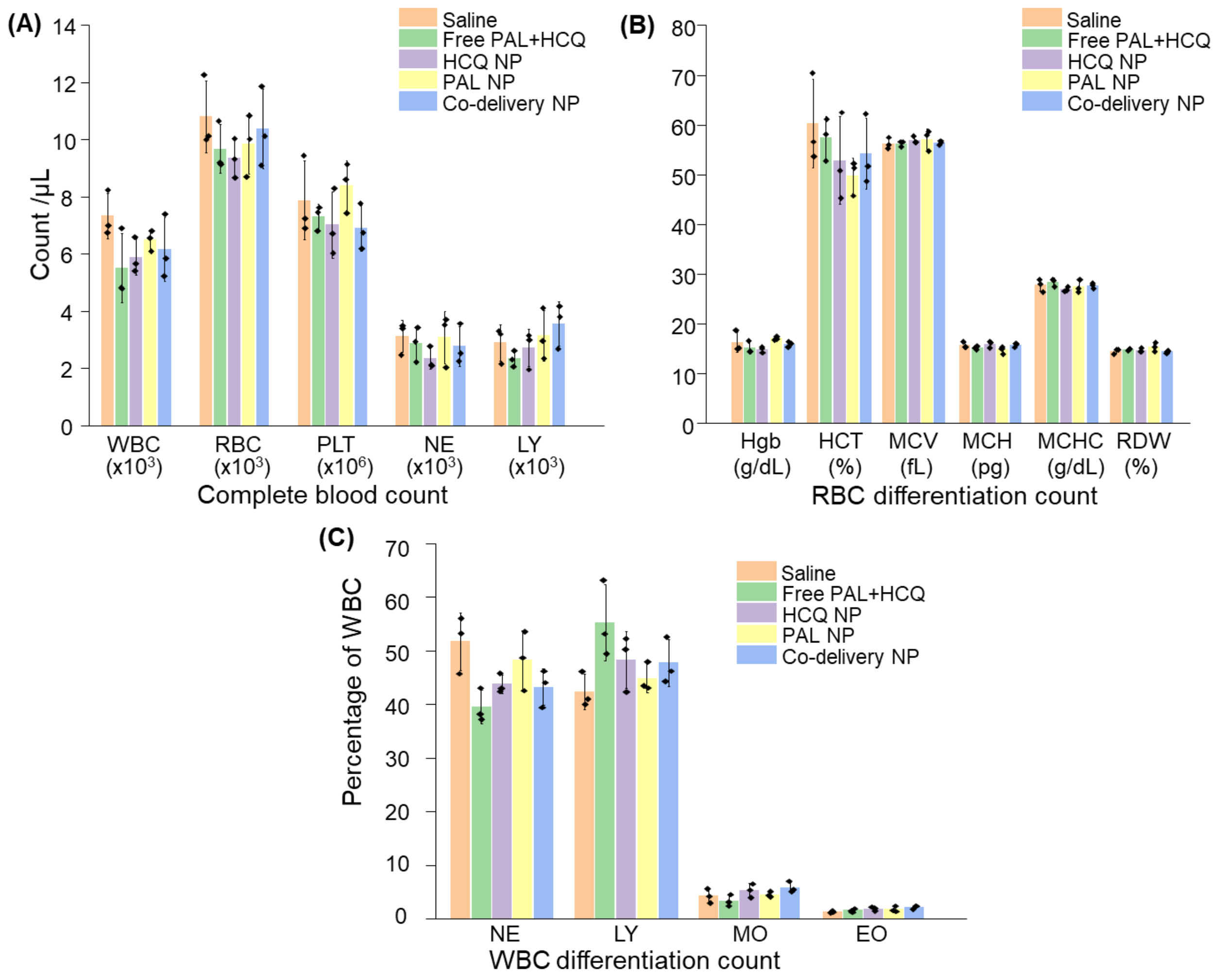

(D)

	ALT	AST	ALP	DBILI	TBILI	GGT	LDH	TP	BUN	CK	CREAT
	(U/L)	(U/L)	(U/L)	(mg/dL)	(mg/dL)	(U/L)	(U/L)	(g/dL)	(mg/dL)	(U/L)	(mg/dL)
Saline	47.5±18.2	123.7±20.2	50.5±6.2	0.6±0.2	0.5±0.1	5.1±1.0	630.1±63.1	4.7±0.2	27.3±2.1	633.2±90.6	0.6±0.1
Free PAL+HCQ	50.1±11.2	178.6±11.7	66.3±8.8	0.4±0.1	0.4±0.1	3.7±0.4	499.0±47.9	4.8±0.5	25.6±3.1	503.2±47.2	1.9±0.5
HCQ NP	34.1±9.5	132.0±19.8	59.8±7.1	0.3±0.1	0.7±0.1	2.9±0.7	465.4±39.3	5.8±0.9	20.1±1.7	501.7±56.3	0.7±0.2
PAL NP	51.7±7.8	162.3±22.3	63.2±10.2	0.8±0.1	0.7±0.2	6.0±0.9	503.7±55.4	5.5±0.3	23.9±2.2	397.9±29.7	0.7±0.1
Co-delivery NP	61.2±13.5	158.9±21.2	66.0±11.3	0.8±0.3	0.4±0.1	6.2±0.9	499.8±38.7	4.8±0.6	22.9±0.7	553.3±43.1	0.9±0.2

(E)

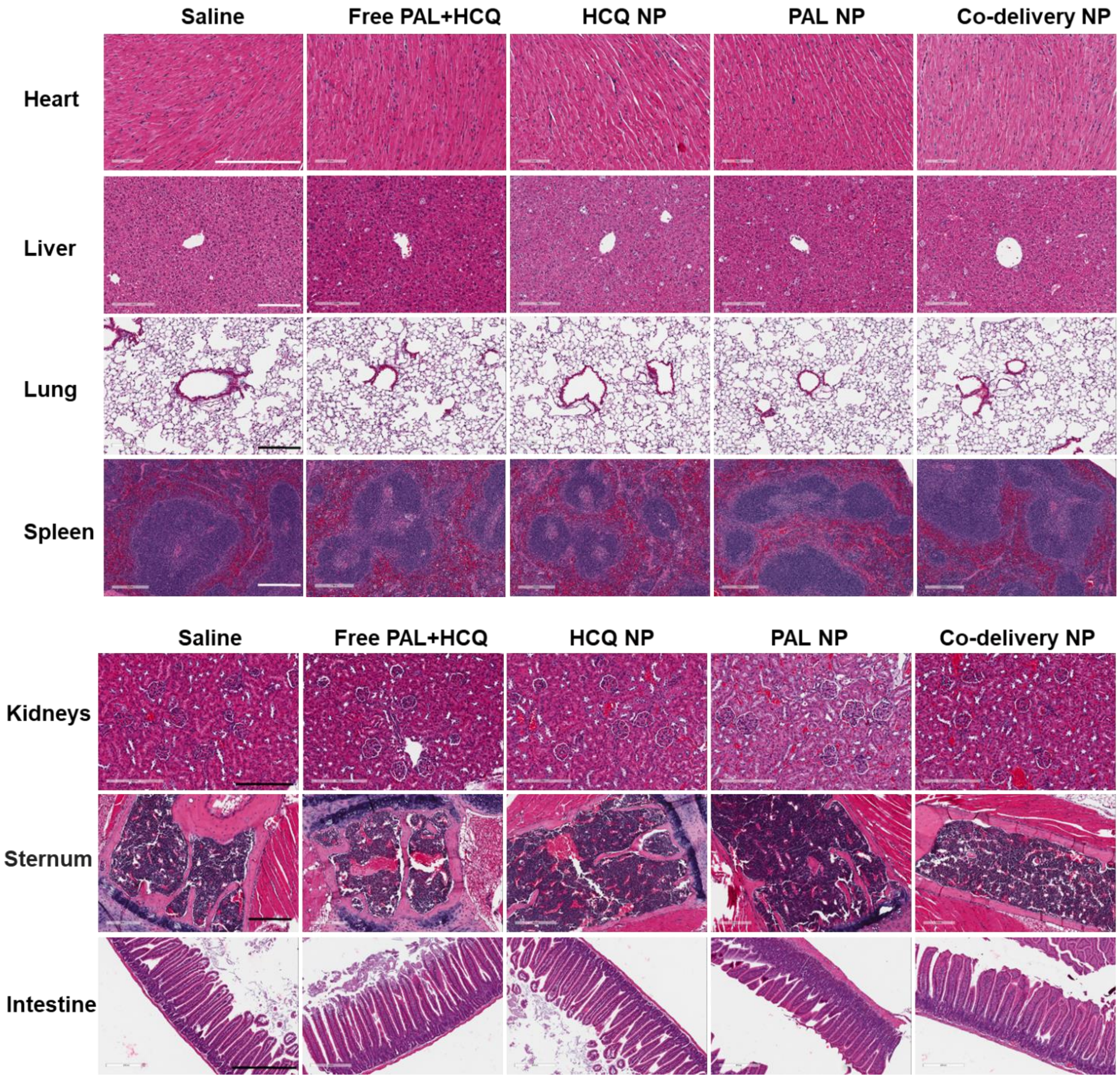

Supplementary Figure 13. IHC staining of pRb and Ki67 on tumor sections from the endpoint of efficacy study as we described in Figure 4D. Data were obtained from one set of independent experiment without repetition. Quantitative evaluation was analyzed by Aperio ImageScope 12.3. Percentages of positive cells were determined by total number of positive events (N_p) divided by total number of all events (N_{total}) in each low-magnification IHC image. Three independent IHC images were analyzed for each group (n=3). Scale bar=200 μm. Data represent mean ± SD, statistical difference was evaluated by one-way ANOVA followed by Tukey's post hoc test (*p<0.05). Consistent with Figure 4E, co-delivery NP exhibit distinct anti-proliferation effect, which can be demonstrated by the significantly reduced expression of both pRb (p=0.018) and Ki67 (p=0.042), compared to PAL NP. Source data are provided as a Source Data file.

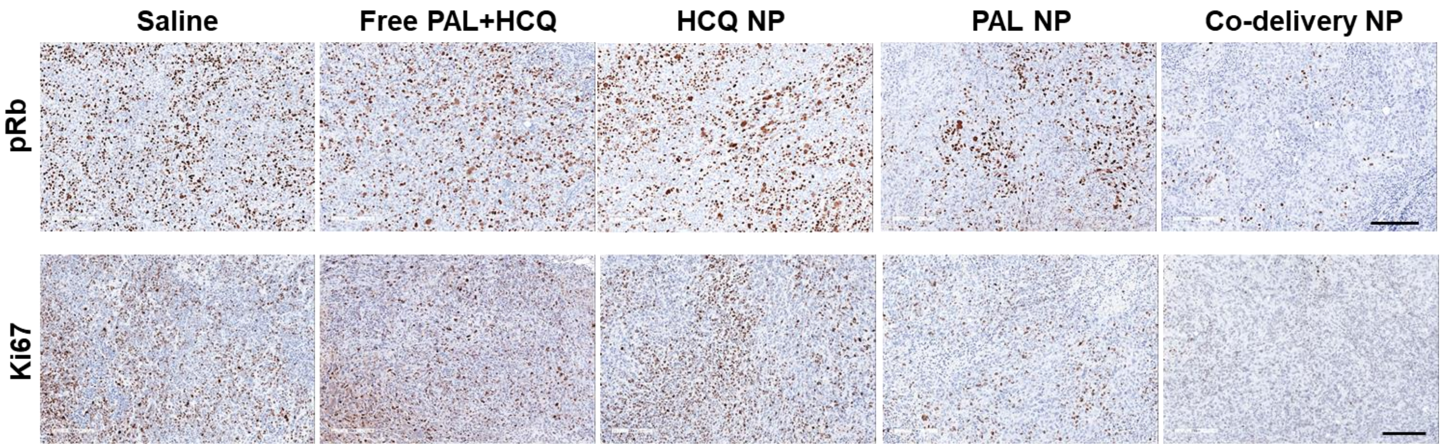

(low magnification)

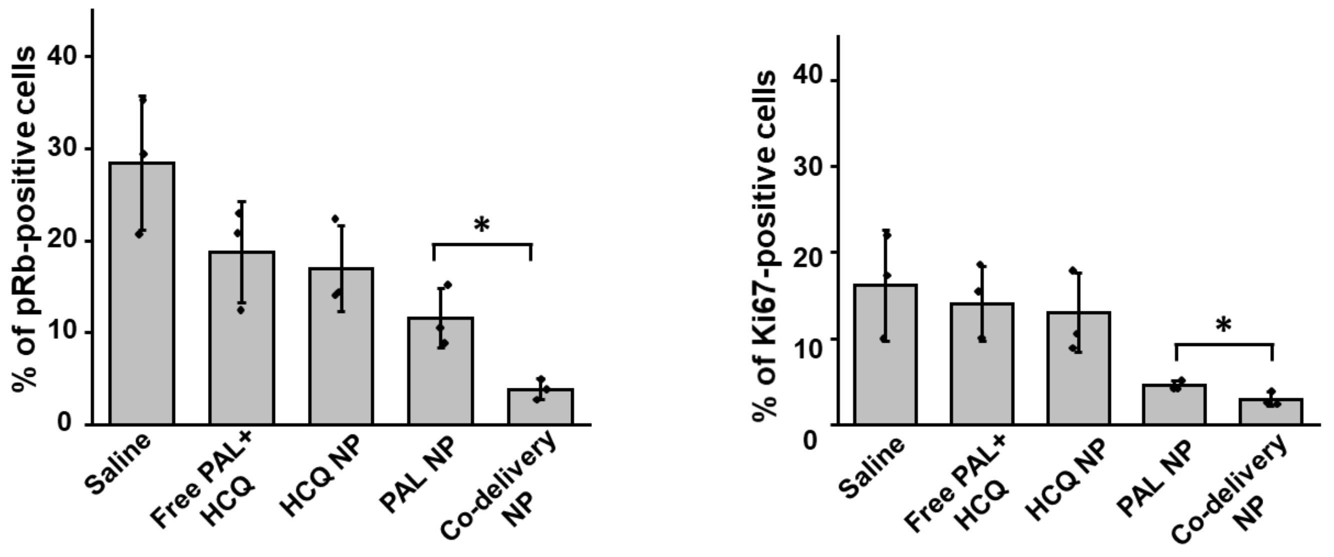

Supplementary Figure 14. IHC staining of cleaved caspase 3 (CC-3) at both high magnification (scale bar=100 μm) and low magnification (scale bar=200 μm) on tumor sections from the same efficacy study as we described in Figure 4D. Data were obtained from one set of independent experiment without repetition. Percentages of positive cells were determined by total number of positive events (N_p) divided by total number of all events (N_{total}) in each low-magnification IHC image. Three independent IHC images were analyzed for each group (n=3). Data represent mean ± SD. The statistical difference was evaluated by one-way ANOVA followed by Tukey's post hoc test. No significant CC-3 expression is induced by co-delivery NP. Source data are provided as a Source Data file.

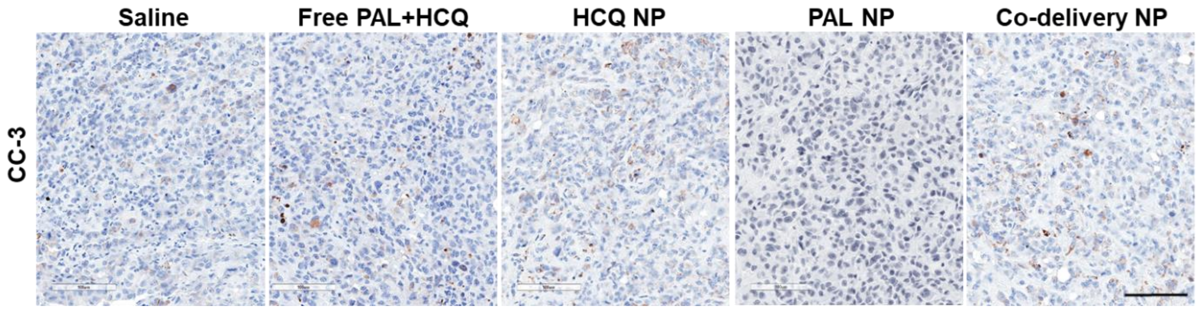

(high magnification)

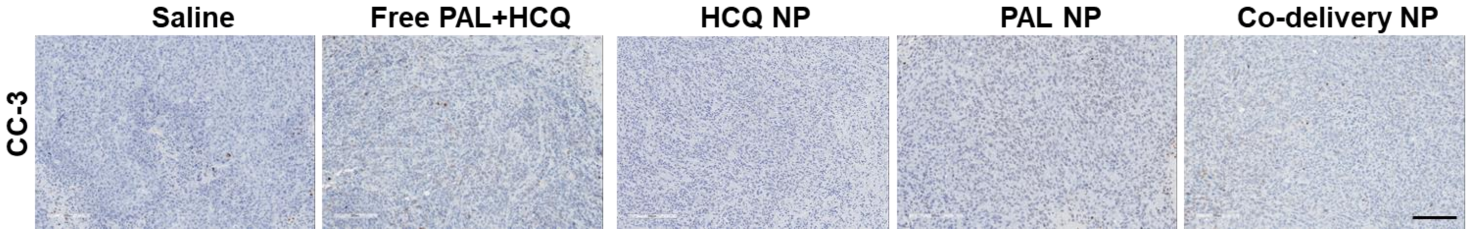

(low magnification)

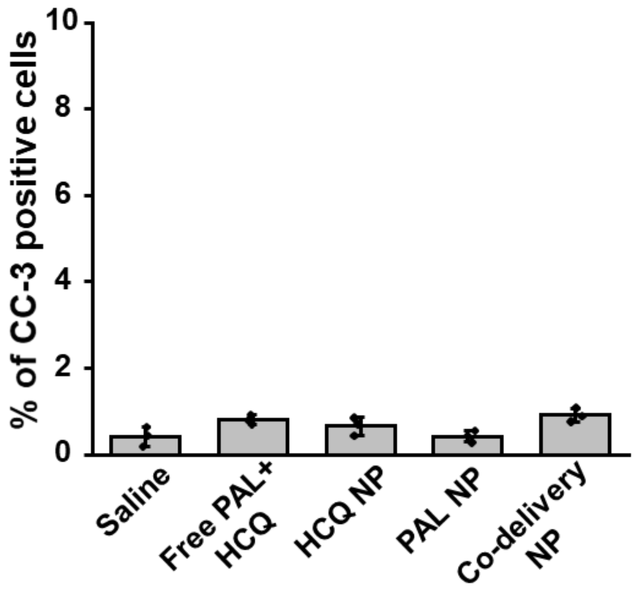

Supplementary Figure 15: Western blot of Bcl-x_L and Mcl-1 in PANC-1 cells treated with co-delivery NP. PANC-1 cells were incubated with co-delivery NP, HCQ NP or PAL NP for 72 h, with equivalent concentration of PAL (2.5 μM) and HCQ (10.8 μM). One of two repetitions with similar results is shown here. Complementary to Figure 5B, the result demonstrates the upregulation of Bcl-x_L and degradation of Mcl-1 by co-delivery NP. Uncropped gel images are provided as a Source Data file.

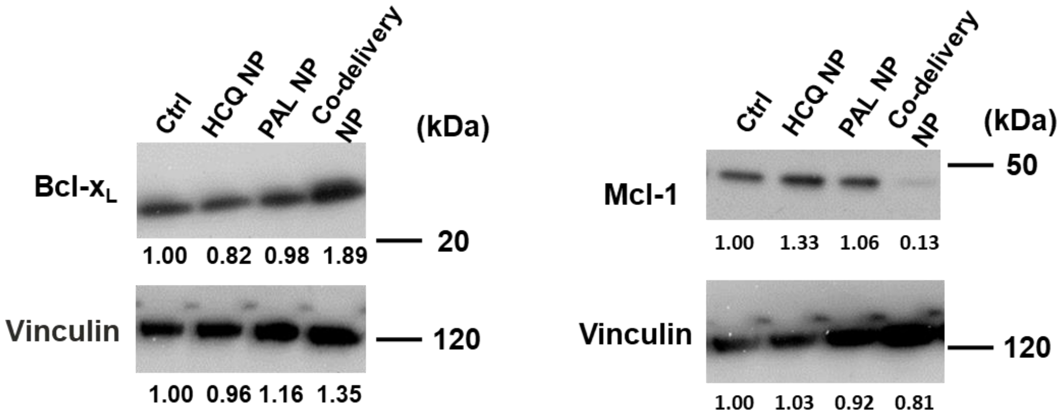

Supplementary Figure 16. IHC staining of Bcl-x_L and Mcl-1 at both high magnification (Scale bar=100 μm) and low magnification (scale bar=200 μm) on tumor sections from the same efficacy study as we described in Figure 4D. Data were obtained from one set of independent experiment without repetition. Three independent IHC images were analyzed for each group (n=3). Data represent mean ± SD, statistical difference was evaluated by one-way ANOVA followed by Tukey's post hoc test (*$p<0.05$). For Bcl-x_L expression, significant difference is detected between co-delivery NP and the remaining treatments, *e.g.* co-delivery NP *versus* free PAL+HCQ (p=0.013). Co-delivery nanoparticles reduce Mcl-1 expression as compared to all the controls, *e.g.* co-delivery NP *versus* Free PAL+HCQ (p=0.010). Source data are provided as a Source Data file.

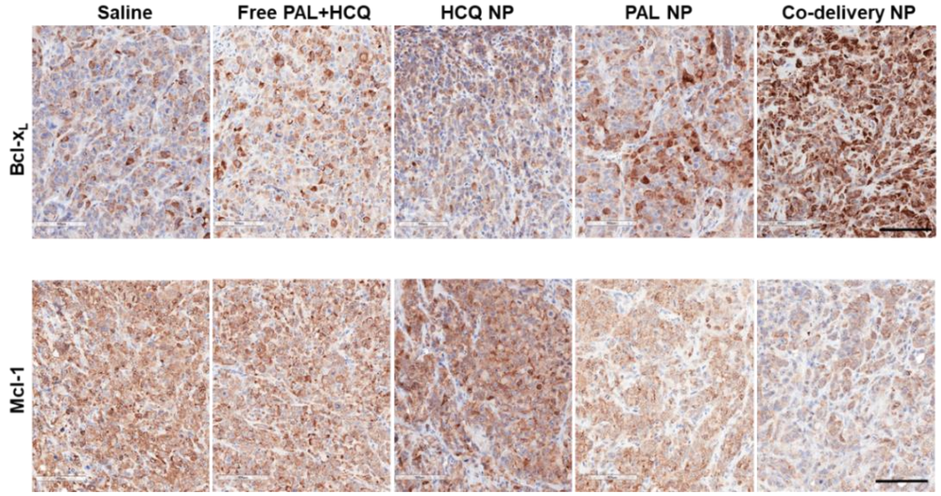

(high magnification)

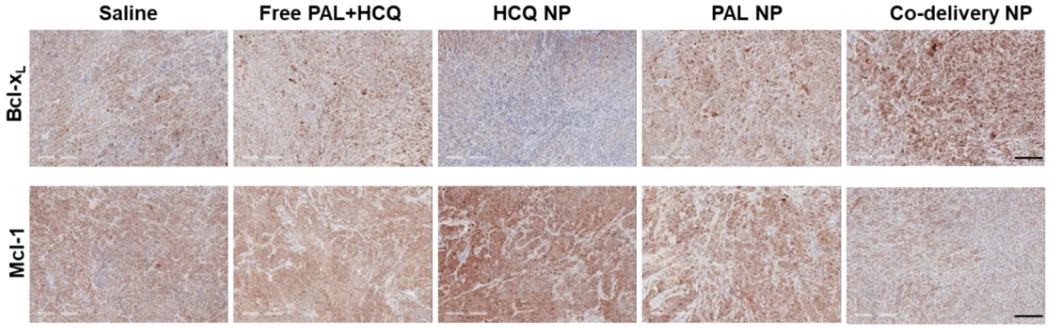

(low magnification)

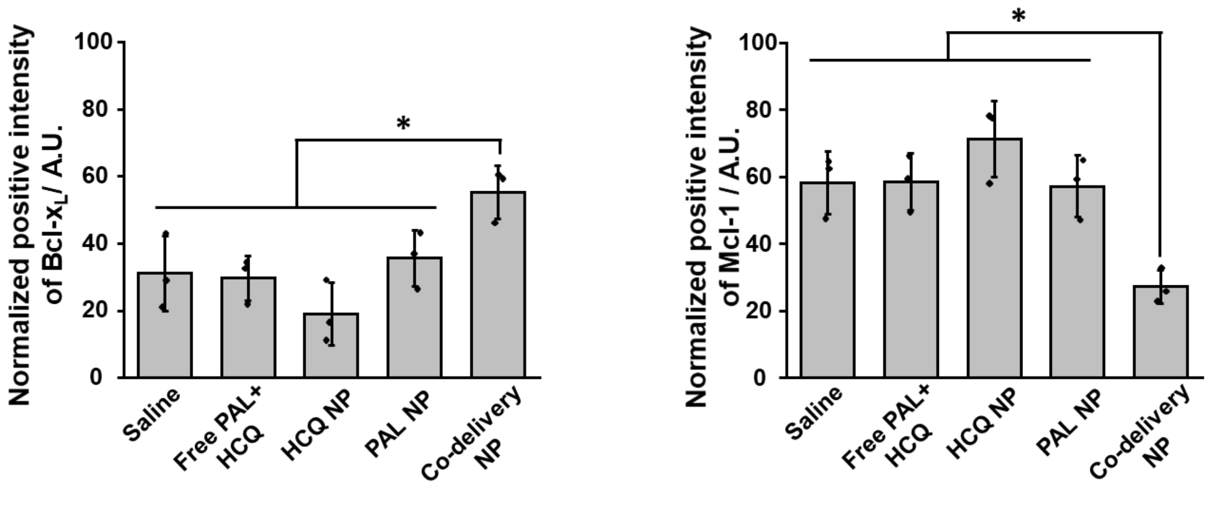

Supplementary Figure 17. Detection of senescence in PDAC cells treated by co-delivery NP. PDAC cells were incubated with indicated treatments for 144 h with equivalent concentration of PAL (2.5 μM) and HCQ (10.8 μM). (A) Senescence-associated beta-galactosidase (SA-β-gal) assay was performed using a commercial kit (Cell Signaling Technology) as per manufacturer's instruction. One of two repetitive experiments with similar results is shown here. Contrast to PAL NP, co-delivery NP do not induce significant senescence in all tested PDAC cell lines. Scale bar=50 μm. (B) RT-PCR detection of senescence associated secretory proteins (SASPs) was performed with RNeasy mini kit (Qiagen) and iTaq™ universal SYBR green one-step kit (BioRad), on ViiA-7 96 well RT-PCR system (Life Technologies). PANC-1 cells were used for the SASP analysis. SASPs gene expression was normalized to a housekeeping gene (GAPDH) and expressed as fold change relative to control. For each group, the results were obtained from cell samples in triplicate (n=3). Data represent mean ± SD, statistical difference was evaluated by one-way ANOVA followed by Tukey's post hoc test (*$p<0.05$). While PAL-NP increase SASP expression, no such finding is observed in the group of co-delivery NP. Primers: Forward GAPDH: 5'-TTAAAAGCAGCCCTGGTGAC-3'; Reverse GAPDH: 5'-CTCTGCTCCTCCTGTTCGAC-3'; Forward IL6: 5'-CTGCAGCCACTGGTTCTGT-3'; Reverse IL6: 5'-CCAGAGCTGTGCAGATGAGT-3'; Forward DCR2: 5'-CCCAGAGGGATGGTCAAGGT-3'; Reverse DCR2: 5'-GTAGTGATAGGGAGAGGCAAGCA-3'; Forward PML: 5'-GCCGACTTCTGGTGCTTTGA-3'; Reverse PML: 5'-GGGTTGGAGCAGAAGATGTTG-3'. Source data are provided as a Source Data file.

(A)

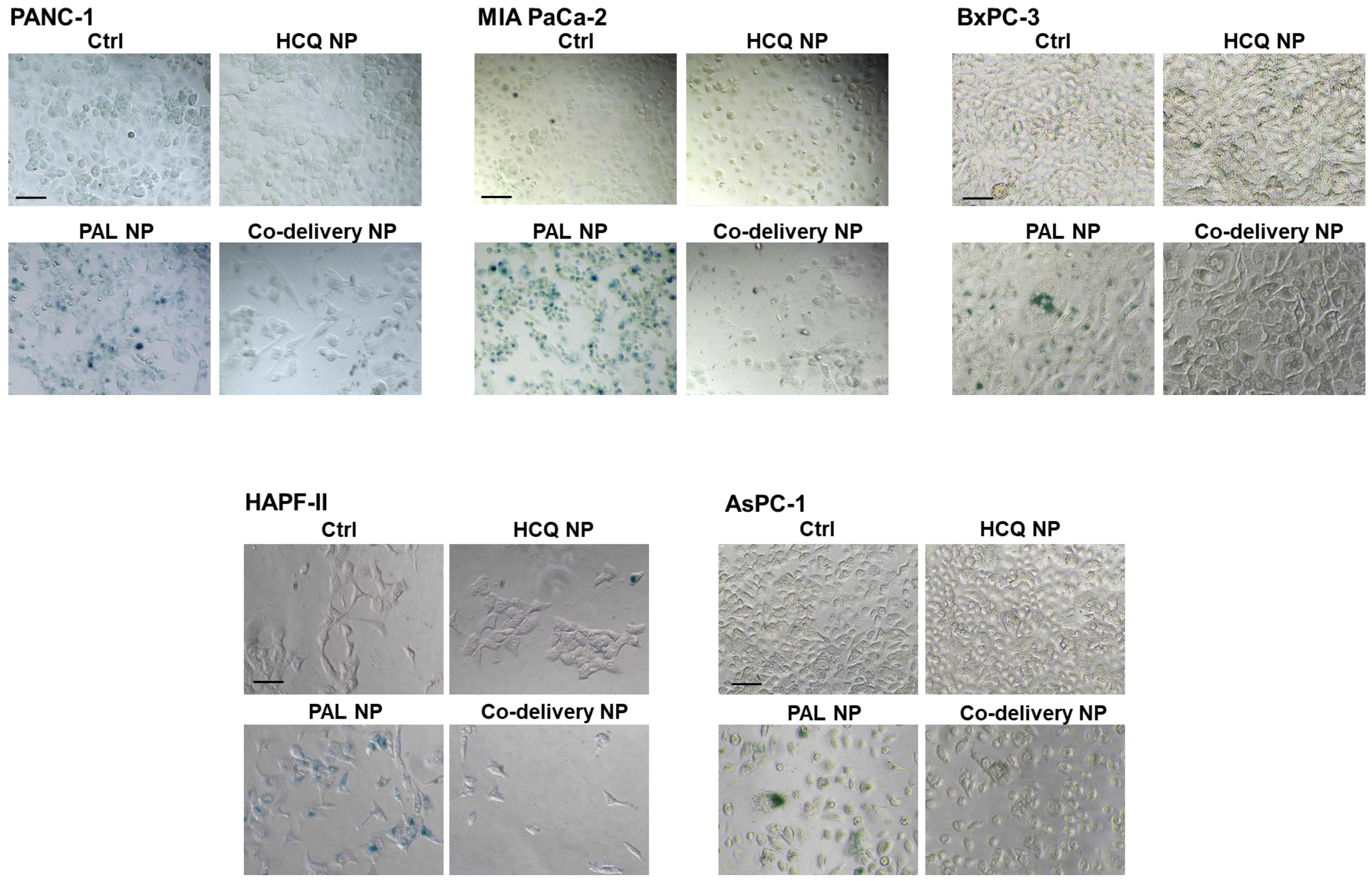

(B)

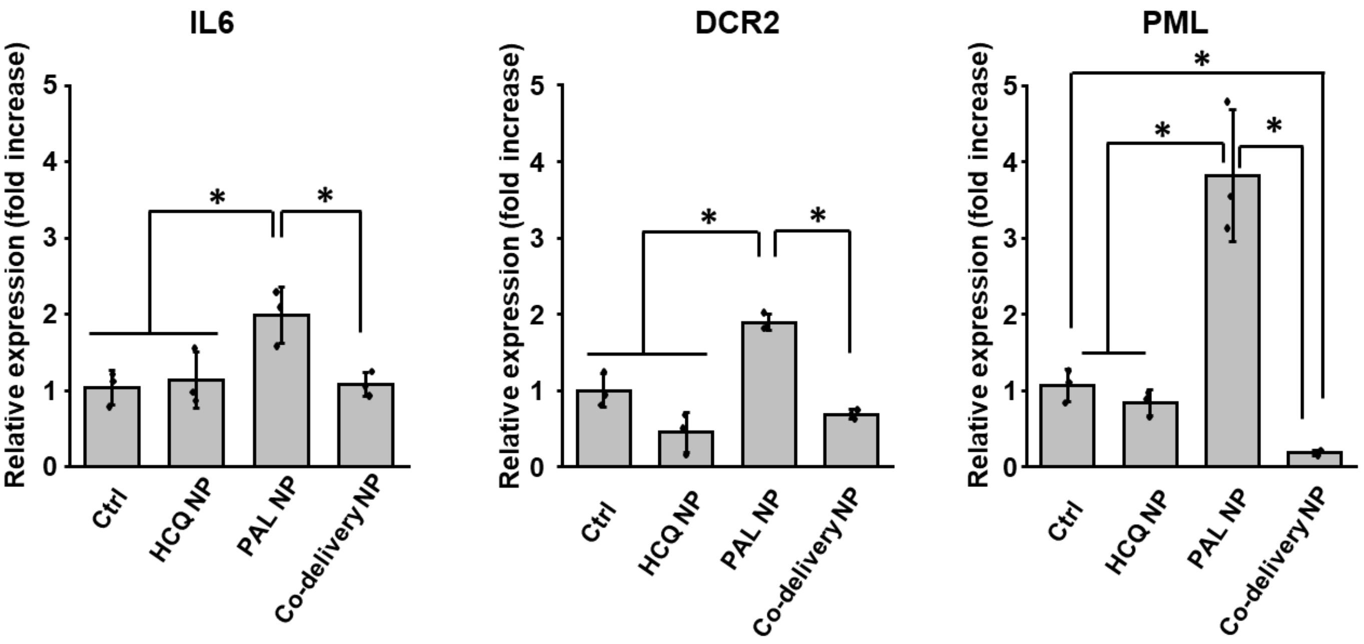
IL6
DCR2
PML
Relative expression (fold increase)
0
1
2
3
4
5
*
Ctrl
HCQ NP
PAL NP
Co-delivery NP

Supplementary Figure 18. Replenishment approach *versus* sequential approach. For the combined use of ABT-737 and co-delivery NP, *in vitro* proliferation study was also performed using a sequential treatment scheme. In brief, PANC-1 cells were treated by co-delivery NP (with equivalent PAL concentration 0-10 μM) from day 0-3. The co-delivery NP were washed off and replaced by ABT-737 (0-10 μM) containing medium for additional 4 days. BrdU proliferation assay was used to determine the cell proliferation on day 7. For each group, the results were obtained from cell samples in triplicate wells (n=3). Data represent mean ± SD, statistical difference was evaluated by one-way ANOVA followed by Tukey's post hoc test (*$p<0.05$). The replenishment approach demonstrates improved anti-proliferation effect than the sequential approach *in vitro* (*e.g.* at 10 μM, $p=0.0026$ and at 0.63 μM, $p=0.0018$). Accordingly, co-delivery NP and ABT-737 were co-administrated in the efficacy study (Figure 5C). Source data are provided as a Source Data file.

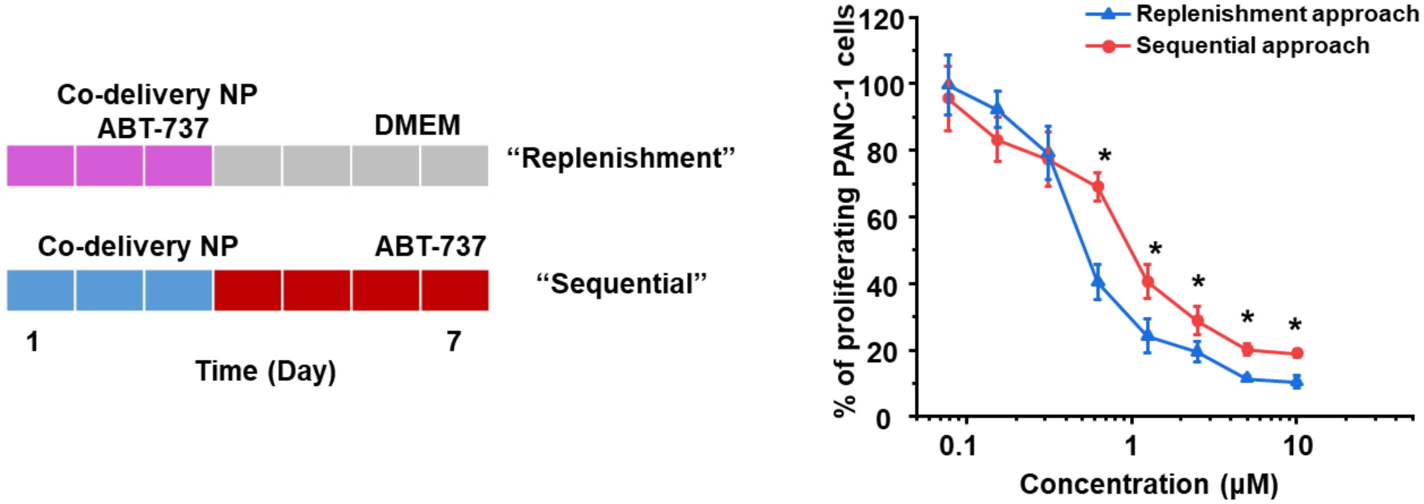

Supplementary Figure 19. Low magnification IHC staining of Ki67 and CC-3 on tumor sections from the endpoint of the efficacy study in Figure 5D. Scale bar=200 μm. Percentage of Ki67 positive or CC-3 positive cells were determined by total number of positive events (N_p) divided by total number of all events (N_{total}) in each low-magnification IHC image (n=3). Percentage of Ki67 positive cells or CC-3 positive cells were then normalized to saline control. Data represent mean ± SD, statistical difference was evaluated by one-way ANOVA followed by Tukey's post hoc test (*p<0.05). By co-administration of ABT-737, co-delivery NP induce persistent anti-proliferation (Ki67) and apoptosis (CC-3) compared to co-delivery NP alone, with p values of 0.013 and 0.00046, respectively. Source data are provided as a Source Data file.

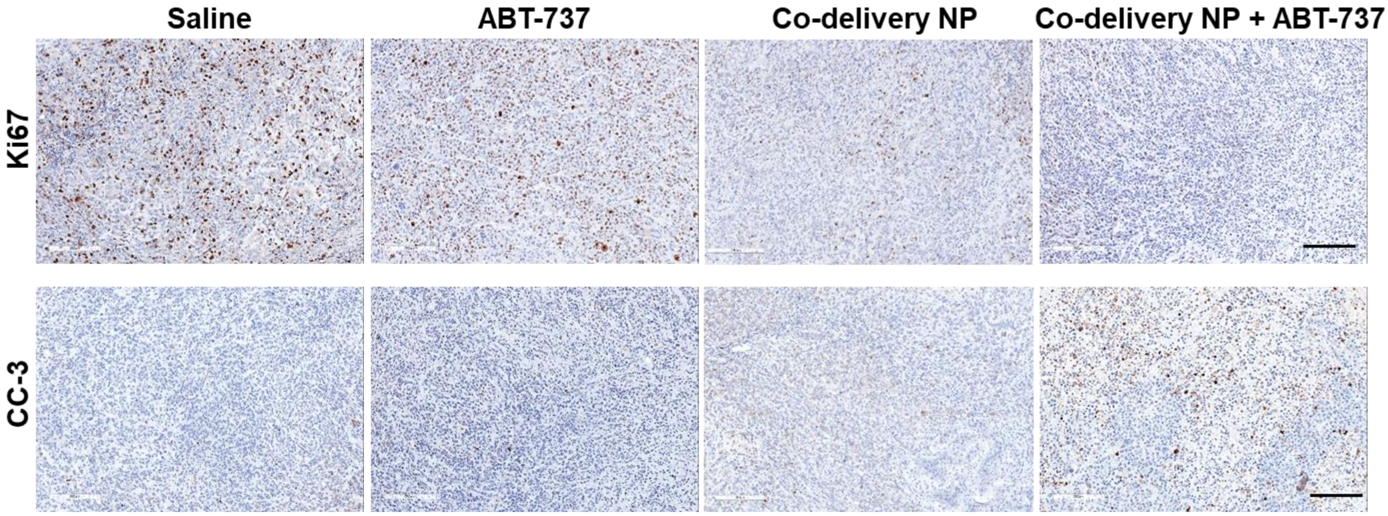

(low magnification)

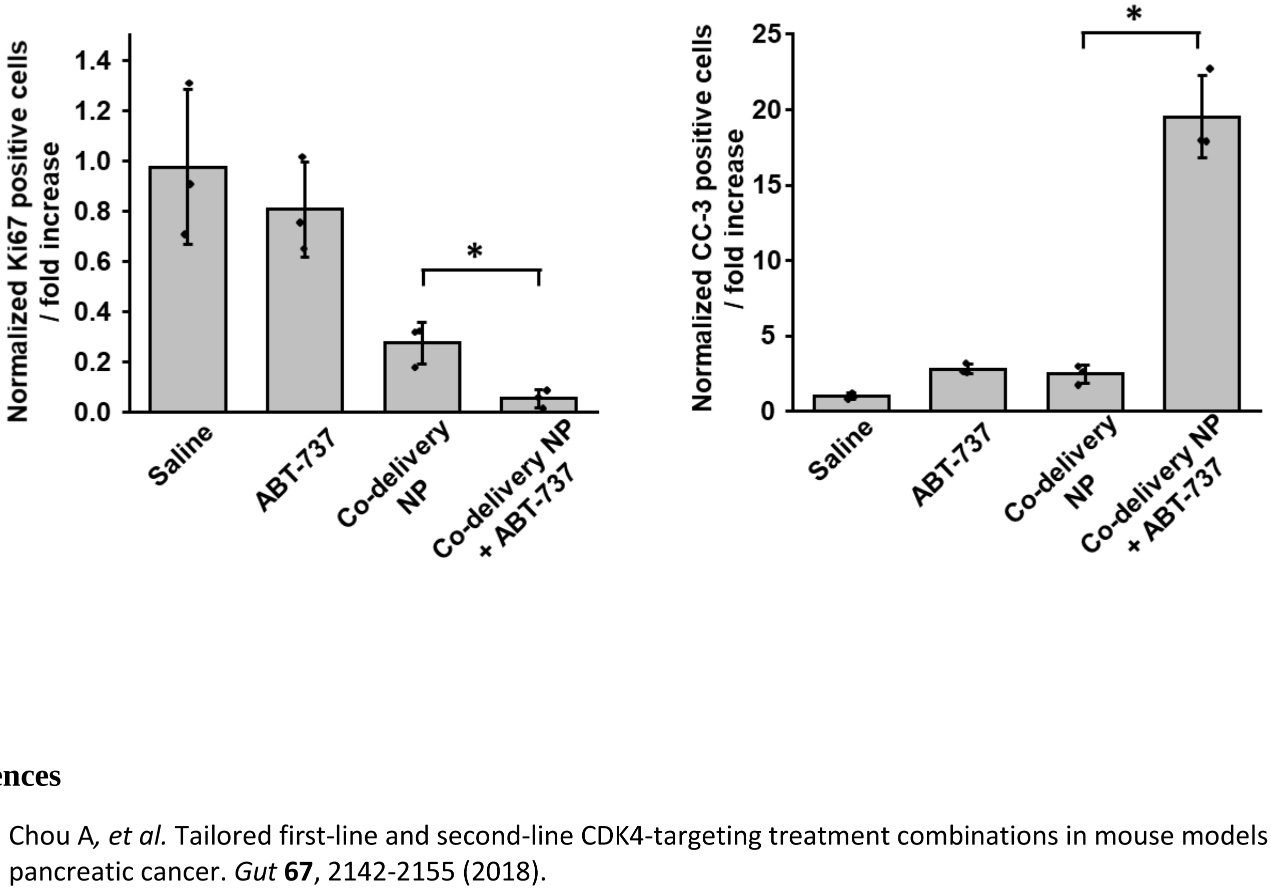

References

1. Chou A*, et al.* Tailored first-line and second-line CDK4-targeting treatment combinations in mouse models of pancreatic cancer. *Gut* **67**, 2142-2155 (2018).
