# Supplementary material for: Use of ratiometrically designed nanocarrier targeting CDK4/6 and autophagy pathways for effective pancreatic cancer treatment
Source: Nat Commun. 2020 Aug 25;11:4249. doi: 10.1038/s41467-020-17996-7 (PMC7447818; doi:10.1038/s41467-020-17996-7)
Supplement: Supplementary file 3 — Reporting Summary [file 41467_2020_17996_MOESM3_ESM.pdf]

nature research

Corresponding author(s): Huan Meng

Last updated by author(s): Jul 15, 2020

Reporting Summary

Nature Research wishes to improve the reproducibility of the work that we publish. This form provides structure for consistency and transparency in reporting. For further information on Nature Research policies, see our Editorial Policies and the Editorial Policy Checklist.

Statistics

For all statistical analyses, confirm that the following items are present in the figure legend, table legend, main text, or Methods section.

n/a	Confirmed	
☐	☒	The exact sample size (n) for each experimental group/condition, given as a discrete number and unit of measurement
☐	☒	A statement on whether measurements were taken from distinct samples or whether the same sample was measured repeatedly
☐	☒	The statistical test(s) used AND whether they are one- or two-sided<br>*Only common tests should be described solely by name; describe more complex techniques in the Methods section.*
☒	☐	A description of all covariates tested
☐	☒	A description of any assumptions or corrections, such as tests of normality and adjustment for multiple comparisons
☐	☒	A full description of the statistical parameters including central tendency (e.g. means) or other basic estimates (e.g. regression coefficient) AND variation (e.g. standard deviation) or associated estimates of uncertainty (e.g. confidence intervals)
☒	☐	For null hypothesis testing, the test statistic (e.g. F, t, r) with confidence intervals, effect sizes, degrees of freedom and P value noted<br>*Give P values as exact values whenever suitable.*
☒	☐	For Bayesian analysis, information on the choice of priors and Markov chain Monte Carlo settings
☒	☐	For hierarchical and complex designs, identification of the appropriate level for tests and full reporting of outcomes
☒	☐	Estimates of effect sizes (e.g. Cohen's d, Pearson's r), indicating how they were calculated

Our web collection on statistics for biologists contains articles on many of the points above.

Software and code

Policy information about availability of computer code

Data collection	Living Image Software v4.5 (IVIS Imaging Systems), LAS X (Leica Confocal SP8), BD FACSDiva™ software v7.0, SpectraMax SoftMax® Pro 7 Software, Gatan DigitalMicrograph GMS3 (TEM/cryoEM), and MassLynx 4.1 software (LCT-Premier ESI-LCMS).
Data analysis	Origin 2018, Aperio ImageScope 12.3, ImageJ v1.5.2, FlowJo v7.6.1, CompuSyn software, and PKSolver.

For manuscripts utilizing custom algorithms or software that are central to the research but not yet described in published literature, software must be made available to editors and reviewers. We strongly encourage code deposition in a community repository (e.g. GitHub). See the Nature Research guidelines for submitting code & software for further information.

Data

Policy information about availability of data

All manuscripts must include a data availability statement. This statement should provide the following information, where applicable:

- Accession codes, unique identifiers, or web links for publicly available datasets
- A list of figures that have associated raw data
- A description of any restrictions on data availability

The data that support the findings of this study are available from the corresponding author upon reasonable request.

Field-specific reporting

Life sciences study design

All studies must disclose on these points even when the disclosure is negative.

Sample size	The sample size was determined based on literature, logistics and/or our experiences. Relevant publications were cited in the manuscript.
Data exclusions	No data was excluded from the analysis.
Replication	Experiments were successfully performed with sufficient animals per group to demonstrate the statistical significance. The number of repeats was defined in each figure legend.
Randomization	Tumor bearing mice were randomly assigned into different treatment groups. The mice were purchased from a major vendor and age-matched.
Blinding	The investigators were not blinded in this preclinical investigation.

Reporting for specific materials, systems and methods

We require information from authors about some types of materials, experimental systems and methods used in many studies. Here, indicate whether each material, system or method listed is relevant to your study. If you are not sure if a list item applies to your research, read the appropriate section before selecting a response.

Materials & experimental systems

n/a	Involved in the study	
☐	☒	Antibodies
☐	☒	Eukaryotic cell lines
☒	☐	Palaeontology and archaeology
☐	☒	Animals and other organisms
☒	☐	Human research participants
☒	☐	Clinical data
☒	☐	Dual use research of concern

Methods

n/a	Involved in the study	
☒	☐	ChIP-seq
☐	☒	Flow cytometry
☒	☐	MRI-based neuroimaging

Antibodies

Antibodies used

Antibodies were used for western blot, immunofluorescence staining and immunohistochemistry staining.
For western blot, the antibodies include: anti-Rb (1:1000, cat#9313, Cell Signaling Technology), anti-phospho-Rb (1:1000, cat#8516, Cell Signaling Technology), anti-β-actin (1:1000, cat#3700, Cell Signaling Technology), anti- SQSTM1/p62(1:1000, cat#ab56416, Abcam), anti-Bcl-xL (1:1000, cat#ab32370, Abcam), anti-Mcl-1 (1:1000, cat#ab32087, Abcam), anti-Vinculin (1:1000, cat#13901, Cell Signaling Technology), anti-Ki67 (1:5000, cat# ab92742, Abcam), and anti-LC3B (1:1000, cat#2775, Cell Signaling Technology). For immunofluorescence staining, the antibodies include anti-phospho-Rb (1:1000, cat#8516, Cell Signaling Technology), anti-SQSTM1/p62 (1:100, cat#ab91526, Abcam), anti-Ki67 (1:500, cat#ab15580, Abcam), anti-cleaved caspase-3(1:400, cat#9664, Cell Signaling Technology), anti-Bcl-2(1:150, cat#ab182858, Abcam), anti-Mcl-1(1:500, cat#ab32087, Abcam), and anti-Bcl-xL (1:500, cat#ab32370, Abcam). For immunohistochemistry staining, we used anti-phospho-Rb (1/200, cat#8516, Cell Signaling Technology), anti-Ki67 (1/100, cat#ab15580, Abcam), anti-cleaved caspase 3 (1/200, cat#9664, Cell Signaling Technology), anti-Mcl-1 (1/200, cat#ab32087, Abcam) and anti-Bcl-xL (1/400, cat#ab32370, Abcam). The secondary antibodies included Alexa Fluor 594 goat anti-rabbit IgG (1:500, cat#A11012, Thermo Fisher Scientific), Alexa Fluor 488 goat anti-rabbit IgG (1:500, cat#A11008, Thermo Fisher Scientific), anti-rabbit HRP-linked IgG (1/1000, cat#7074, Cell Signaling Technology), and anti-mouse HRP-linked IgG (1/1000, cat#7076, Cell Signaling Technology).

Validation

Above-mentioned antibodies were selected based on the literature and/or manufactures' instruction. Anti-SQSTM1/p62 (cat#ab56416, Abcam, https://www.abcam.com/sqstm1--p62-antibody-autophagosome-marker-ab56416.html),anti-Bcl-xL (cat#ab32370, Abcam, https://www.abcam.com/bcl-xl-antibody-e18-ab32370.html),anti-Ki67 (cat# ab92742, Abcam), anti-Ki67 (cat#ab15580, Abcam, https://www.abcam.com/ki67-antibody-epr3610-ab92742.html),anti-Bcl-2(cat#ab182858, Abcam, https://www.abcam.com/bcl-2-antibody-epr17509-ab182858.html) were validated using knockout cell lines by the manufacturers and are extensively used in the scientific community. Anti-Mcl-1 (cat#ab32087, Abcam, https://www.abcam.com/mcl1-antibody-y37-ab32087.html), anti-SQSTM1/p62 (cat#ab91526, Abcam, https://www.abcam.com/sqstm1-p62-antibody-ab91526.html), anti-phospho-Rb (cat#8516, Cell Signaling Technology), anti-β-actin (cat#3700, Cell Signaling Technology),anti-Vinculin (cat#13901, Cell Signaling Technology), anti-LC3B (cat#2775, Cell Signaling Technology, https://www.cellsignal.com/products/primary-antibodies/lc3b-antibody/2775), anti-phospho-Rb (cat#8516, Cell Signaling Technology, https://www.cellsignal.com/products/primary-antibodies/phospho-rb-ser807-811-d20b12-xp-rabbit-mab/8516), anti-cleaved caspase 3 (cat#9664, Cell Signaling Technology, https://www.cellsignal.com/products/primary-antibodies/cleaved-caspase-3-asp175-5a1e-rabbit-mab/9664) have been validated by their manufacturers and are extensively used in the scientific community. Anti-Rb (cat#9313, Cell Signaling Technology, https://www.cellsignal.com/products/primary-antibodies/rb-d20-rabbit-mab/9313) has been validated by manufacturers using SimpleChIP® Enzymatic Chromatin IP Kits.

Eukaryotic cell lines

Policy information about cell lines

Cell line source(s)	PANC-1, MIA PaCa-2, BxPC3, HPAF-II were provided by ATCC. AsPC-1 was provided by Dr. Nanping Wu at UCLA and originally purchased from ATCC. The immortalized KPC cell line derived from a spontaneously developed tumor in a transgenic KrasLSL G12D/+; Trp53LSL R172H/+; Pdx-1-Cre mouse was originally provided by Dr. Andrew Lowy at UC San Diego.
Authentication	All the cells in this study were obtained from standard commercial sources with authentication or isolated according to reported researches.
Mycoplasma contamination	All cell lines tested negative for mycoplasma.
Commonly misidentified lines (See ICLAC register)	No commonly misidentified cell lines were used in the study.

Animals and other organisms

Policy information about studies involving animals; ARRIVE guidelines recommended for reporting animal research

Laboratory animals	Athymic Balb/c nude mice were provided by Charles River Laboratory. 6-8 weeks old female mice were used in the experiments. The protocols are approved by the Chancellor's Animal Research Committee at UCLA and include standard operating procedures for animal housing (filter-topped cages; room temperature at 23 ± 2 °C, 60% relative humidity; 12 h light/ 12 h dark cycle) and hygiene status (autoclaved food and acidified water).
Wild animals	n/a
Field-collected samples	n/a
Ethics oversight	Mice were housed in specific-pathogen-free conditions according to the institution guideline. The mice were monitored on daily basis.

Note that full information on the approval of the study protocol must also be provided in the manuscript.

Flow Cytometry

Plots

Confirm that:

- [x] The axis labels state the marker and fluorochrome used (e.g. CD4-FITC).
- [x] The axis scales are clearly visible. Include numbers along axes only for bottom left plot of group (a 'group' is an analysis of identical markers).
- [x] All plots are contour plots with outliers or pseudocolor plots.
- [x] A numerical value for number of cells or percentage (with statistics) is provided.

Methodology

Sample preparation	To probe cell cycle, PDAC cells incubated with free drugs or drug loaded nanoparticles were harvested and washed with PBS. Cells were fixed with 4% paraformaldehyde for 10 min at room temperature. Cells were washed with PBS and incubated with Tali cell cycle solution (Thermo Fisher) that contains propidium iodide (PI), RNase A, and Triton X-100, for 30 min in dark. To obtain single cell suspension, the cells were passed through a 70 µm cell strainer. Cells were then subjected to flow cytometry for cell cycle analysis.<br>To characterize ROS production, PDAC cells incubated with free drug or drug loaded nanoparticles were stained with the CellROX® Green flowcytometry kit (Thermo Fisher) as per manufacturer's instruction for 30 minutes at 37°C. Cells were washed with PBS and harvested. Cells were passed through a 70 µm cell strainer then subjected to flow cytometry for ROS detection.
Instrument	BD™ LSR II (BD Bioscience) in the UCLA Jonsson Comprehensive Cancer Center flow cytometry core
Software	BD FACSDiva™ software v7.0, FlowJo 7.6.1
Cell population abundance	No cell sorting was involved in this study.
Gating strategy	The gating strategy used was forward and side scatter gating to remove debris and other events of noninterest while preserving cells based on size and/or complexity.

- [x] Tick this box to confirm that a figure exemplifying the gating strategy is provided in the Supplementary Information.
